# Mapping of Craniofacial Traits in Outbred Mice Identifies Major Developmental Genes Involved in Shape Determination

**DOI:** 10.1371/journal.pgen.1005607

**Published:** 2015-11-02

**Authors:** Luisa F. Pallares, Peter Carbonetto, Shyam Gopalakrishnan, Clarissa C. Parker, Cheryl L. Ackert-Bicknell, Abraham A. Palmer, Diethard Tautz

**Affiliations:** 1 Department of Evolutionary Genetics, Max Planck Institute for Evolutionary Biology, Plön, Germany; 2 Department of Human Genetics, University of Chicago, Chicago, Illinois, United States of America; 3 Center for Musculoskeletal Research, University of Rochester, Rochester, New York, United States of America; 4 Department of Psychiatry and Behavioral Neuroscience, University of Chicago, Chicago, Illinois, United States of America; Georgia Institute of Technology, UNITED STATES

## Abstract

The vertebrate cranium is a prime example of the high evolvability of complex traits. While evidence of genes and developmental pathways underlying craniofacial shape determination is accumulating, we are still far from understanding how such variation at the genetic level is translated into craniofacial shape variation. Here we used 3D geometric morphometrics to map genes involved in shape determination in a population of outbred mice (Carworth Farms White, or CFW). We defined shape traits via principal component analysis of 3D skull and mandible measurements. We mapped genetic loci associated with shape traits at ~80,000 candidate single nucleotide polymorphisms in ~700 male mice. We found that craniofacial shape and size are highly heritable, polygenic traits. Despite the polygenic nature of the traits, we identified 17 loci that explain variation in skull shape, and 8 loci associated with variation in mandible shape. Together, the associated variants account for 11.4% of skull and 4.4% of mandible shape variation, however, the total additive genetic variance associated with phenotypic variation was estimated in ~45%. Candidate genes within the associated loci have known roles in craniofacial development; this includes 6 transcription factors and several regulators of bone developmental pathways. One gene, *Mn1*, has an unusually large effect on shape variation in our study. A knockout of this gene was previously shown to affect negatively the development of membranous bones of the cranial skeleton, and evolutionary analysis shows that the gene has arisen at the base of the bony vertebrates (Eutelostomi), where the ossified head first appeared. Therefore, *Mn1* emerges as a key gene for both skull formation and within-population shape variation. Our study shows that it is possible to identify important developmental genes through genome-wide mapping of high-dimensional shape features in an outbred population.

## Author Summary

Formation of the face, mandible, and skull is determined in part by genetic factors, but the relationship between genetic variation and craniofacial development is not well understood. We demonstrate how recent advances in mouse genomics and statistical methods can be used to identify genes involved in craniofacial development. We use outbred mice together with a dense panel of genetic markers to identify genetic loci affecting craniofacial shape. Some of the loci we identify are also known from past studies to contribute to craniofacial development and bone formation. For example, the top candidate gene identified in this study, *Mn1*, is a gene that appeared at a time when animals started to form bony skulls, suggesting that it may be a key gene in this evolutionary innovation. This further suggests that *Mn1* and other genes involved in head formation are also responsible for more fine-grained regulation of its shape. Our results confirm that the outbred mouse population used in this study is suitable to identify single genetic factors even under conditions where many genes cooperate to generate a complex phenotype.

## Introduction

Understanding the evolutionary processes that have generated and maintained morphological diversity in nature is a long-standing goal in biology. The cranium and mandible of vertebrates is a good example of such diversity. The fact that the cranial and mandible bones have to be integrated with the brain and sensory systems, as well as with the respiratory and digestive systems, makes this structure a prime example of both high integration and high evolvability.

Although information about genes and developmental pathways involved in shape determination keeps accumulating, we are far away from understanding the genotype-phenotype map translating genetic variation into craniofacial shape variation [1]. To approach this question, here we aim to identify the genetic factors underlying such morphological differences.

Previous experimental work has explored the genetic basis of craniofacial variation in a range of species, including Darwin’s finches [2–4], cichlids [5,6], dogs [7–9], and mice [10–16]. There has also been some recent work on natural facial variation in humans [17–20]. Much of this work has been made possible by developments in geometric morphometrics, which provide the techniques for quantifying subtle shape variation [21]. Combined with increasing availability of genomics resources for mice, this has made possible genome-wide studies of natural shape variation in mice [12].

Early work in mice has focused mainly on the mandible. This is a well-established model for the study of complex traits because mandible shape can be approximated in 2 dimensions [22,23]. Several quantitative trait loci (QTL) studies have investigated mandible shape variation, and have identified several genomic regions underlying 2D variation in this trait, mostly in crosses of inbred laboratory strains [13,24,25]. Although the skull has received less attention due to its higher complexity and the difficulty of defining appropriate phenotypes (2D vs 3D), recently Burgio et al. [15], Pallares et al. [12], and Maga et al. [10] have successfully identified genomic regions underlying 3D skull variation in mice. In our previous study [12], we used genome-wide association (GWAS) based on natural recombinants from a hybrid zone between two subspecies of the house mouse. This enabled us to identify candidate skull and mandible shape loci with much higher resolution than conventional QTL studies in mice (e.g. [10]).

Here, we approach the question from a micro-evolutionary perspective by analyzing within-population shape variation. The utility of studying phenotypic variation at the within-population level is well acknowledged, as it permits one to focus on within-species genetic contributions to phenotypic variation [26]. We use a population of “Carworth Farms White” (CFW) outbred mice, whose suitability for genome-wide mapping was previously described [27–29]. Recently developed genomic resources for this population allow for QTL mapping on autosomal chromosomes (see Methods). The CFW mice were originally derived from a small number of Swiss mice, and have been maintained for dozens of generations as an outbred colony with a large breeding population that avoids crosses between closely related individuals [27,30,31]. Importantly, the mice used in this study show little evidence for population stratification or cryptic relatedness, which simplifies the analysis and interpretation of genetic variation contributing to quantitative traits. The high number of recombination events in the history of this population has resulted in small linkage blocks, which, together with the above mentioned features, result in high mapping resolution [27].

## Results

We estimated heritability of craniofacial shape, and assessed support for craniofacial shape and size QTLs at 80,027 autosomal SNPs in 592–720 mice. Skull shape is represented as a 132-dimension vector (coordinates of 44 3D landmarks), and mandible shape is represented as a 39-dimension vector (coordinates of 13 3D landmarks). To make the shape data suitable for QTL mapping, we extracted principal components (PCs) that explain the most variance in skull and mandible shape. Specifically, skull shape was represented by the first 22 PCs that capture 84% of the skull shape variation, and mandible shape was represented by the first 21 PCs accounting for 94% of the variation in mandible shape.

### Heritability

**Fig 1:**
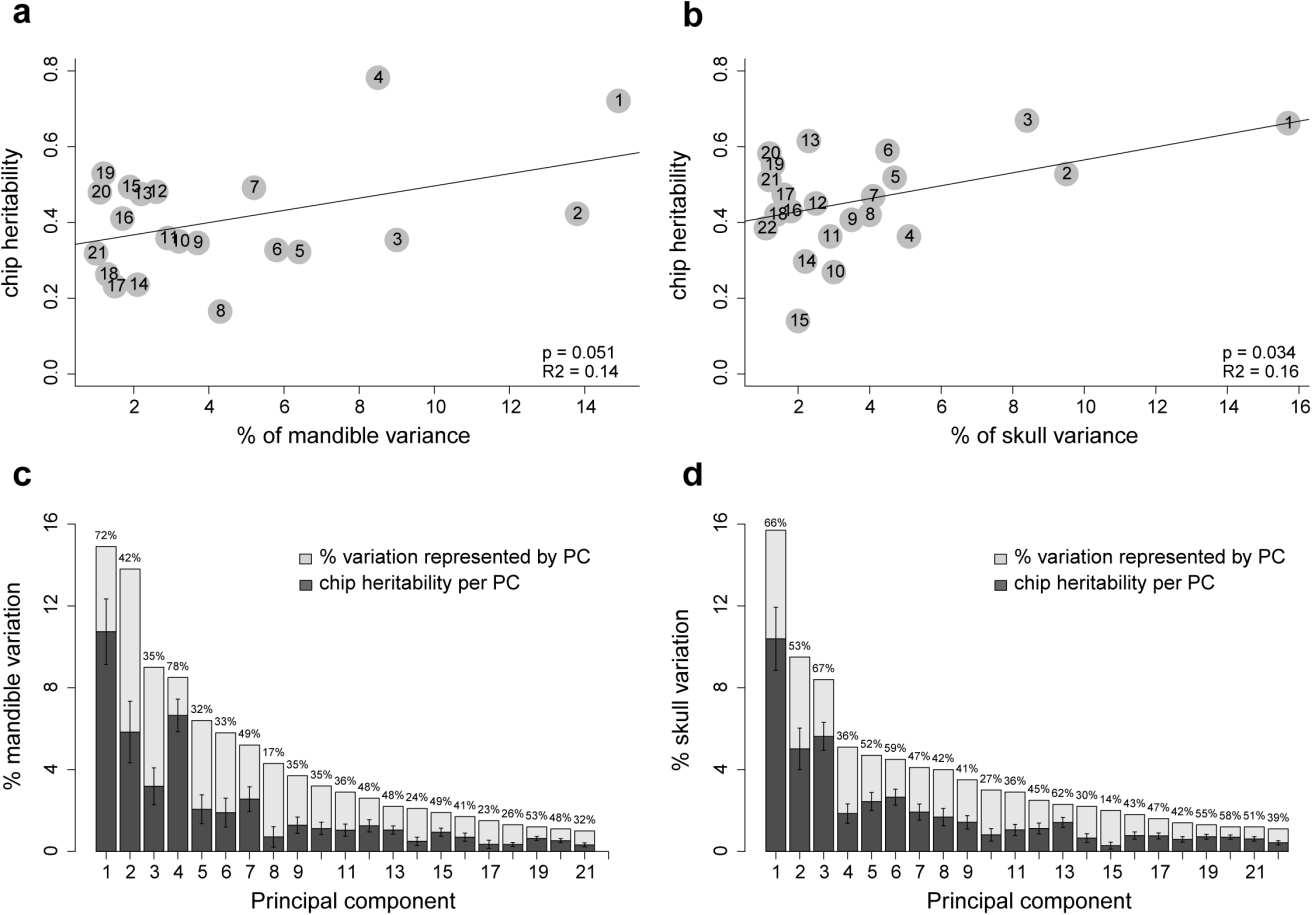
SNP heritability of individual PCs. Correlation between SNP heritability and proportion of variation explained by the PC is shown for (a) mandible and (b) skull. Grey dots represent PCs. In (c) and (d), numbers above the bars indicate the proportion of each bar that is colored dark grey, this is the SNP heritability of each PC. The error bars give standard error of the SNP heritability estimates.

Heritability of each skull and mandible PC was calculated using the standard additive polygenic model (S2 and S3 Tables and Fig 1). The heritability values we report here are “SNP heritability” [32]; that is, the estimate of the proportion of phenotypic variance explained by all available SNPs. All skull and mandible PCs exhibit substantial contributions from the additive genetic variance component; only two PCs have heritability values lower than 20%. Mandible size has SNP heritability of 36.4% (95% confidence interval 16.4–56.4), and skull size of 35.4% (15–55.8). To summarize the heritability for mandible and skull shape with a single statistic, we calculated a weighted average of the SNP heritability of individual PCs (see Methods and Fig 1C and 1D). These “total heritability” values are 43.6% for mandible shape and 42.4% for skull shape. This statistic is equal to the proportion of the bar chart that is shaded dark gray in Fig 1C and 1D. We also checked whether the proportion of total phenotypic variation explained by each PC is correlated with our SNP heritability estimates. This correlation is not strong, but significant; mandible, r^2^ = 0.14, p-value = 0.034; skull, r^2^ = 0.16, p-value = 0.034 (Fig 1A and 1B).

### Chromosomal partition of the variance

**Fig 2:**
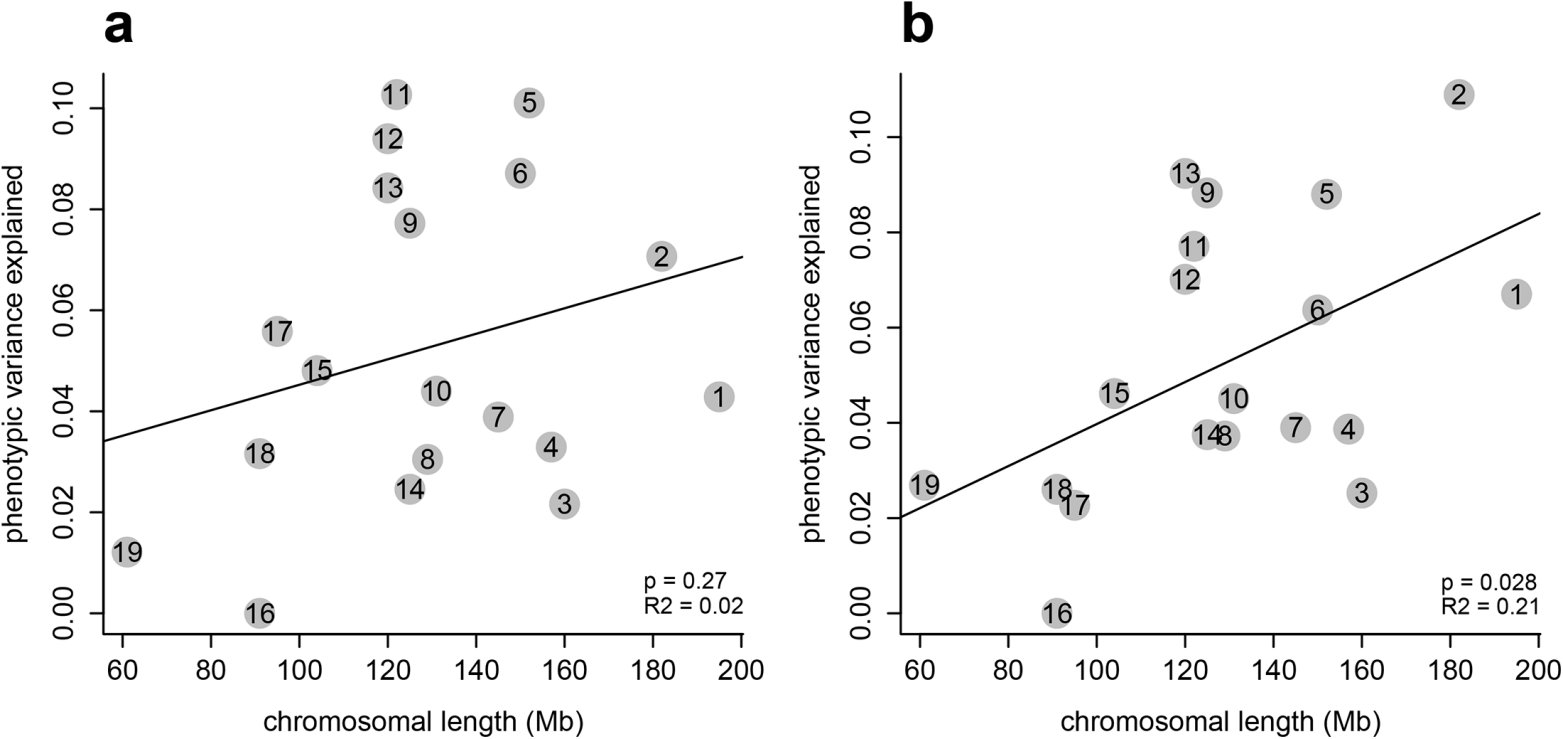
Chromosomal partition of the variance. These plots compare the contribution of each chromosome to (a) mandible and (b) skull shape variation, and how these contributions correlate with chromosome length (in Mb).

Partitioning the variance by chromosome shows almost all chromosomes contribute to shape variation (Fig 2). We find also a correlation with chromosome size, as expected, but this is only statistically significant for mandible shape (Fig 2B). In our previous study we found a highly significant correlation for both, mandible and skull shape [12]. The weaker correlation in the present study is likely due to lower and somewhat uneven marker coverage, which is in itself not strongly correlated with chromosome length (S2 Fig). In particular, chromosome 16 is underrepresented with respect to marker coverage; the fact that this chromosome contributes very little to the phenotypic variance (Fig 2) could be due to technical limitations regarding SNP identification, or to this chromosome indeed harboring little variation.

### Genomic regions associated with craniofacial size and shape

**Fig 3:**
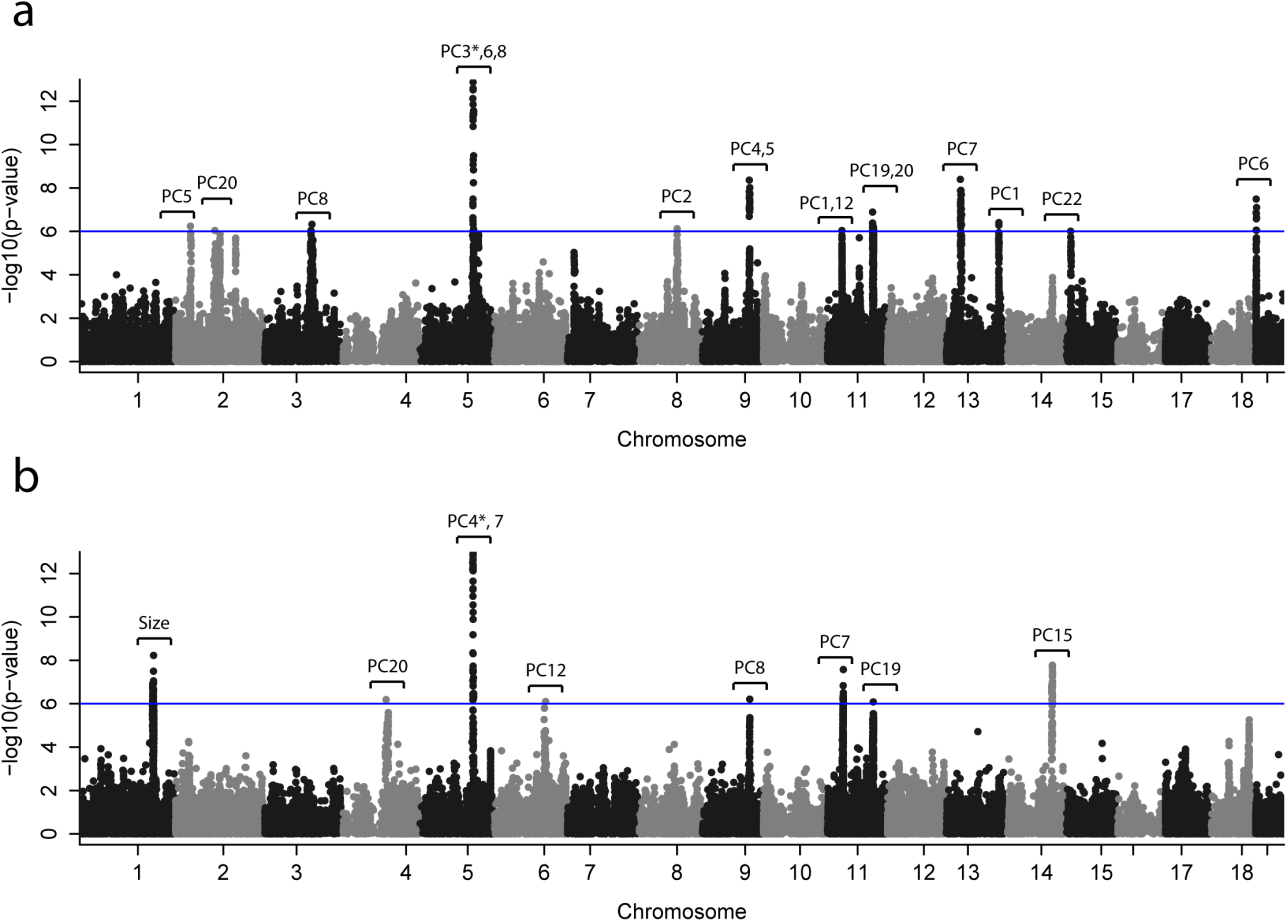
Genome-wide scans for (a) mandible and (b) skull. This plot shows *p*-values for association with craniofacial phenotypes—22 skull shape PCs, 21 mandible shape PCs and centroid size—at 80,027 candidate SNPs. Since only the smallest *p*-values are visible from this plot, *p*-values for individual PCs are drawn in separate plots; see S9 and S10 Figs. The associated phenotype (PC or centroid size) is indicated for each QTL. The blue line represents an approximate genome-wide significance threshold, 1e-6 (-log(p) = 6); the actual threshold we used to determine significance of the p-values is different for each phenotype (average–log(p) = 6.05, min = 5.95, max = 6.16). *To improve visualization, the *p*-value shown in the Figure is larger than the actual p-value; the actual p-values are PC4* -log(p) = 26.6, PC3* -log(p) = 14.8.

**Fig 4:**
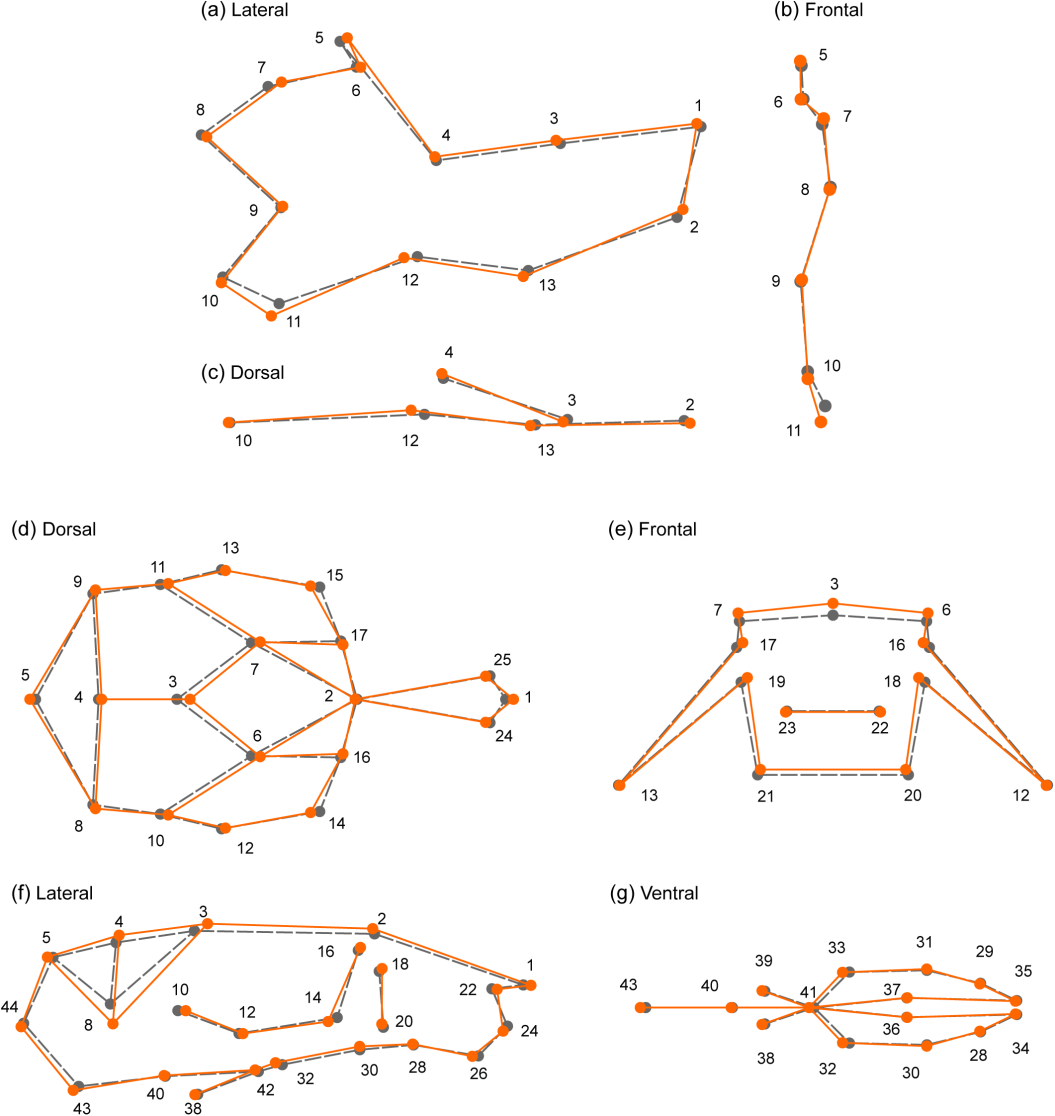
Changes in mandible (a-c) and skull (d-g) shape associated with the SNPs of largest effect. SNP rs33702397 explains 2% of skull shape variation, and rs33614268 explains 1.4% of mandible shape variation. The sample mean shape is depicted in grey (dotted line). In orange (continuous line) is the mean shape associated to the SNP effect, scaled 10x. For mandible, (a) lateral, (b) frontal, and (c) dorsal views are shown. For skull, (d) dorsal, (e) frontal, (f) lateral, and (g) ventral views are shown. Dots and numbers represent relevant landmarks for each view.

Out of the 22 PCs used to map skull shape, 12 PCs had at least one significant QTL; and out of the 20 PCs used in the mapping of mandible shape, 7 PCs had a significant QTL (see Fig 3 and Tables 1 and 2). 17 QTLs were identified for skull shape variation (Table 1), and eight QTLs for mandible shape variation (Table 2). One QTL was associated with mandible centroid size, and no QTLs were identified for skull centroid size. The shape traits associated with the “peak SNPs” (SNPs with lowest p-value) are depicted in Figs 4 and S4–S8.

**Table 1:** SNPs associated with skull phenotypes (PCs). Columns of the table show the SNP with the lowest p-value per QTL, its base-pair position, p-value calculated in GEMMA, and the proportion of total skull shape variation explained by the SNP (%varSkull).

**Region**	**Skull**	**Chr**	**Pos**	**SNP**	**p-value**	**%varSkull**	**Candidate genes**
1	PC1	11	32367260	rs258942042	9.13E-07	0.75	*Sh3pxd2b*
2	PC1	13	110231696	rs245694506	3.93E-07	0.86	*Rab3c*, *Plk2*, *Pde4d*
3	PC2	8	80889309	rs228570244	7.54E-07	0.62	*Gab1*, *Inpp4b*
4	PC3	5	111328046	rs33702397	2.18E-27	2.05	*Mn1*
5† **	PC4	9	99713529	rs30491142	9.69E-09	0.44	*Cldn18*
6**	PC5	2	33284278	rs27194486	5.74E-07	0.35	*Lmx1b*
7† **	PC5	9	98588137	rs13466556	4.35E-09	0.56	*Foxl2*
8*	PC6	5	111626960	rs254983846	1.53E-08	1.27	*Mn1*
9*	PC6	19	4165856	rs37378594	3.24E-08	0.48	*-*
10† *	PC7	13	31734894	cfw-13-31734894	4.00E-09	0.53	*Foxf2*, *Foxc1*
11† *	PC8	3	98931976	rs30352013	4.71E-07	0.38	*Tbx15*
12*	PC8	5	110918274	rs227631022	2.63E-09	1.15	*Mn1*
13	PC12	11	32423285	rs26862534	9.48E-07	0.40	*Sh3pxd2b*
14*	PC19	11	95634099	rs26992385	8.83E-07	0.20	*-*
15	PC20	2	83096089	rs46747509	9.08E-07	0.61	*Itgav*
16*	PC20	11	94881746	rs50079241	1.29E-07	0.47	*Col1a1*, *Dlx3*
17*	PC22	15	11384042	rs31584944	9.85E-07	0.25	*Npr3*

Principal component (PC). %varSkull is the proportion of variation in skull shape explained by the SNP. This value was calculated in a multivariate regression of shape on the SNP genotype. The candidate genes were identified based on their role in bone morphogenesis (see methods). Empty cells (-) mean no compelling candidate gene emerged. The total set of genes in the QTL regions is shown in S4Table

† Regions that overlap with Maga *et al* 2015

* Regions that overlap with Attanasio *et al* 2014 using a window of 500Kb around the focal SNP

** Regions that overlap with Attanasio *et al* 2014 using a window of 1Mb around the focal SNP

**Table 2:** SNPs associated with mandible phenotypes (PCs). Columns of the table show the SNP with the lowest p-value per QTL, its base-pair position, p-value calculated in GEMMA, and the proportion of total skull shape variation explained by the SNP (%varMand).

**Region**	**Mandible**	**Chr**	**Pos**	**SNP**	**p-value**	**%varMand**	**Candidate genes**
1*	PC4	5	111018365	rs33217671	1.66E-15	1.28	*Mn1*
2*	PC7	5	111426493	rs33614268	7.63E-10	1.44	*Mn1*
3*	PC7	11	35295119	rs28219152	2.66E-08	0.44	*-*
4† **	PC8	9	99595168	rs29977169	6.18E-07	0.27	*Cldn18*
5	PC12	6	107312800	rs36343125	7.93E-07	0.22	*-*
6	PC15	14	98935309	rs237064333	1.68E-08	0.22	*Klf5*
7‡ **	PC19	11	96261688	rs233696367	8.25E-07	0.31	*Hoxb cluster*
8†	PC20	4	90510654	rs221759350	6.48E-07	0.26	*-*
9*	Centroid size	1	153481175	rs32618422	5.96E-09	5.08	*-*

Principal component (PC). Centroid size (CS). %varMand is the proportion of variation in mandible shape explained by the SNP. This value was calculated in a multivariate regression of shape on the SNP genotype. The candidate genes were identified based on their role in bone morphogenesis (see methods). Empty cells (-) mean no clear candidate gene emerged. The total set of genes in the QTL regions is shown in S5 Table

† Regions that overlap with Maga *et al* 2015

‡ Region that overlap with Pallares *et al* 2014

* Regions that overlap with Attanasio *et al* 2014 using a window of 500Kb around the focal SNP

** Regions that overlap with Attanasio *et al* 2014 using a window of 1Mb around the focal SNP

In some cases multiple QTLs were found in the same chromosome (chr2, 5, 9, 11, 13) but associated with different PCs (Fig 3). Four QTLs were associated with more than one PC; interestingly, two of them were associated with PCs from skull and mandible (chr5 and chr9).

**Fig 5:**
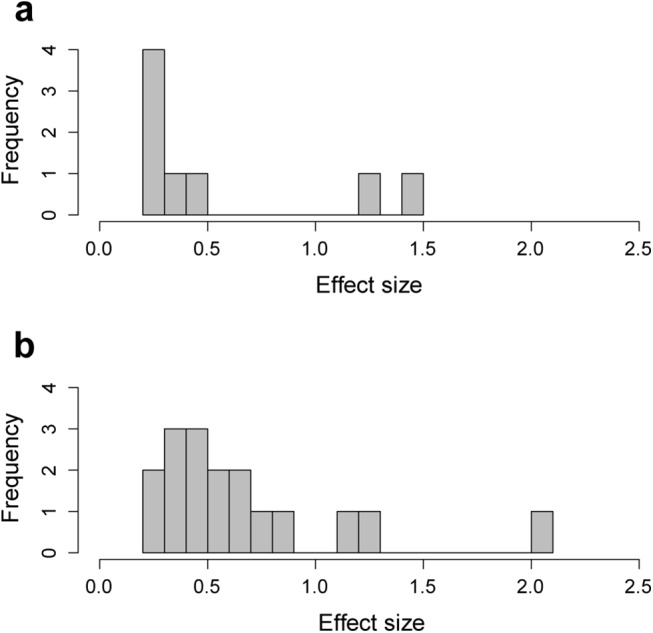
Effect size of peak SNPs (lowest p-value in each QTL) associated with (a) mandible and (b) skull shape. Together the peak SNPs explain 11.4% of variation in skull shape, and 4.4% of variation in mandible shape.

Together the 17 QTLs identified for skull shape explain 11.4% of skull variation. The 8 QTLs for mandible shape together explain 4.4% of mandible variation. The effect size of individual SNPs ranges from 0.02 to 1.13% of the total phenotypic variation (Fig 5 and Tables 1 and 2). The single QTL found for mandible size explains 4.1% of size variation.

### Candidate genes

We compiled a list of 115 protein-coding genes within the craniofacial shape QTLs (S4 and S5 Tables). For most of the regions compelling candidate genes could be identified based on previously reported craniofacial phenotypes or previous evidence for a role in bone morphogenesis. Table 3 lists the functional information available for these genes. Most candidate genes listed in this table are transcription factors or known regulators of developmental signaling cascades.

**Table 3:** Previously published findings supporting involvement of candidate genes in craniofacial phenotypes, in alphabetic order by gene.

Gene	Biochemical function	Developmental function	Mutant phenotype	Human disease association
*Cldn18*	structural component of tight junctions	expressed in osteoblasts [33]; regulates bone resorption and osteoclast differentiation via the RANKL signaling pathway [34]	decreased total body bone mineral density, trabecular bone volume, and cortical thickness [35]	
*Col1a1*	extracellular matrix protein	main component of connective tissues	shows decreased bone volume/tissue volume and reduced trabecular number; exhibits mechanically weak, brittle, fracture-prone bones [36]	osteogenesis imperfecta, a human syndrome characterized by bone fragility; subjects also show craniofacial alterations and deficient osteogenesis [37,38]
*Dlx3*	transcription factor with homeobox domain	regulates adult bone mass and remodeling [39,40]	branchial arch specification and craniofacial defects [39,41]	tricho-dento-osseos syndrome (TDO) in humans, characterized by increased bone mineral density, craniofacial defects, and abnormal teeth and hair [42]
*Foxc1*	transcription factor with forkhead domain	interacts with BMP signaling and *Msx2* to control calvarial bones osteogenesis [43–45] and with Fgf8 to regulate the patterning of the mammalian jaw [46]	congenital hydrocephalus with calvaria bones absent [47]	Axenfeld-Rieger syndrome, includes among other defects abnormalities in teeth and jaw [48]
*Foxl2*	transcription factor with forkhead domain	among other functions, is also active in cranial neural crest cells and cranial mesodermal cells [49]	muscular and skeletal craniofacial malformations [49,50]	blepharophimosis, ptosis, epicanthus inversus syndrome (BPES) characterized by eyelid and craniofacial malformations and ovarian failure [51]
*Gab1*	adaptor molecule with pleckstrin domain	involved in intracellular signaling cascades of EGFR and FGFR and cytokine receptors [52]; regulates osteoblast maturation and mineralization in long bones in mice [53]	embryonic lethal [52]; disruption of its expression in osteoblasts leads to decreased trabecular bone mass with a reduced bone formation rate and a decreased bone resorption [53]	
*Inpp4b*	phosphatase involved in phosphatidylinositol signaling pathways	represses osteoclast differentiation by regulation of the transcription factor *Nfatc1* that beside others, regulates *Itgav* [54,55]	bone loss and osteoporosis [54,55]	BMD variation in pre-menopausal women [54,55]
*Itgav*	integrin family of transmembrane proteins	heterodimer Itgav-Itgb3 is characteristic of osteoclasts, regulating its apoptosis and the process of bone resorption [56]	various phenotypes, including cleft palate [57]	
*Klf5*	transcription factor with Krüppel-like zinc finger domain	regulates the commitment of ES cells to mesoderm lineage [58], and the epithelial-mesenchymal transition [59]	affects tooth development [60]; when overexpressed calvaria bones are absent and mandible is underdeveloped [61]	
*Lmx1b*	transcription factor with homeobox domain	involved in a variety of developmental processes, including limbs, brain, kidney, eye, and calvarial bones [62]	multiple calvarial defects [63]	Nail-patella syndrome (NPS) including limb defects [63]
*Mn1*	transcriptional activator	modifies vitamin D [64] and vitamin A receptor mediated transcription [65] in the context of bone formation and regulates osteoblast development [64,66]	craniofacial defects affecting exclusively membranous bones in the skull [65]	involved in craniofacial deformations [67] and palate cleft syndromes [67,68]
*Npr3*	natriuretic peptide receptor	among other functions, it isinvolved in differentiation and proliferation of bone cells [69,70]	skeletal-overgrowth syndrome with endochondral ossification defects [69,71]	
*Rab3c*	regulatory GTPase	regulates vesicular trafficking in the cell, is expressed in mouse calvaria osteoblast and is thought to play a role in bone mineralization [72]	in cell culture studies it regulates the formation of the ruffled membrane, the resorptive organelle of the osteoclast [73]	
*Pde4d*	phosphodiesterase specific for cAMP degradation	expressed in calvaria osteoblasts [74]; some splice variants regulate BMP-induced bone formation [74,75]	regulates osteoblast differentiation in vitro by degrading cAMP [74,75]	Acrodysostosis, skeletal syndrome including nasal hypoplasia and skull deformities [76]. BMD variation in humans [77]
*Sh3pxd2b*	substrate of Src tyrosine kinase	involved in EGF signaling pathway [78] and the formation of podosomes, which are thought to contribute to tissue invasion and matrix remodeling	craniofacial and skeleton malformations in mice [79–81]	syndromes with craniofacial deformities, Frank-Ter Haar syndrome [82], and Borrone dermato-cardio-skeletal syndrome [83]
*Tbx15*	transcription factor with T-box domain	involved in early endochondral bone development in prehypertrophic chondrocytes of cartilaginous templates [84]	general reduction of bone size and changes of bone shape [84]; droopy-eared mutation in mice [85,86]	Cousin syndrome including craniofacial dysmorphism [87]

## Discussion

The number of regions identified in this study, together with the high mapping resolution in the CFW mouse population, demonstrates the feasibility of mapping within-population variation of complex traits like craniofacial shape. Our results also contribute novel biological insights into the genetic architecture and heritability of craniofacial shape in mice.

### Genetic architecture of craniofacial traits

#### Craniofacial shape

Our results support the notion of a highly polygenic architecture for craniofacial shape in mice. Data derived from other approaches also support this conclusion [11,12]. Such a highly polygenic architecture is expected to facilitate evolutionary modulations and transitions since micro-evolutionary changes at the population level can easily become subject of positive selection and their combined effect can generate large changes in the phenotype.

In total, 17 genomic regions were associated with skull shape, and 8 regions were associated with mandible shape; together they explain 11.4% and 4.4% of the total skull and mandible shape variation, respectively. The total SNP heritability was estimated to be 43.6% for skull shape and 42.4% for mandible shape based on models of polygenic variation for the individual PCs. Although reducing the genetic contribution of a complex, multivariate trait to a single number is necessarily an oversimplification, this nonetheless suggests that the majority of the additive genetic variation is not captured by the SNPs that crossed the significant threshold as defined in this study. This, together with the small effect size of individual loci suggests that the number of loci contributing to the fine-tuning of shape are, at least, in the order of hundreds. We expect that this hidden variation would become apparent with larger sample sizes or in other genetic contexts.

We found little overlap in the regions associated with skull and mandible (Table 1). Given the shared developmental origin of the mandible and some parts of the skull, more overlap would have been expected. However, since it appears that the loci identified by genome-wide association analysis constitute only a small proportion of the real number of functionally relevant loci, it is possible that the lack of overlap reflects the difficulty of identifying strong support for genetic variants contributing to highly complex traits.

Principal components representing small (e.g. PC20) as well as large (e.g. PC1) proportions of the total phenotypic variation were found to associate with genetic variants (Table 1). This pattern was also found previously [12,88]. SNP heritability estimates showed that all PCs included in this study have moderate to high additive genetic variation (S2 and S3 Tables and Fig 1) and therefore associations with genomic regions are expected regardless of the amount of phenotypic variation represented by individual PCs. Mapping approaches that do not rely on principal component analysis show that vectors different from PCs are associated with QTLs (see Maga et al. [10]). The fact that many different dimensions of shape variation associate with genetic variation would be an expected consequence of the highly polygenic architecture of craniofacial shape traits.

#### Craniofacial size

Only one genomic region was significantly associated with mandible size, while no significant associations were found for skull size. Previous studies found up to 23 QTLs associated with mandible size variation [24,25,89], and seven QTLs for skull size variation [10]. Most of these studies are based on mouse lines with a specific contrast in size, i.e. the lines had been selected for large or small size. In our study using a wild-derived population of mice we did not find significant associations with craniofacial size either [12]. Given the large additive genetic variance of craniofacial size in the CFW mice used here, as well as in wild mice [12], the absence of specific associations suggests that the effect size of loci involved in size variation is very small, even smaller than the effect of loci controlling shape, and therefore the power of these two studies was not enough to detect them.

### Heritability of morphological traits

The total heritability estimated in this study, ~43% for craniofacial shape and ~36% for craniofacial size, corresponds to SNP heritability estimates. In humans, SNP heritability is considered an underestimate of the narrow sense heritability because it does not take into consideration rare alleles [90]. However, in the CFW population used here, rare alleles are expected to be uncommon due to the bottleneck when the population was started, the limited number of generations since the bottleneck, and the modest effective population size. Thus, SNP heritability estimates in this population may be closer to narrow sense heritability. Using a population of wild derived mice and a 3D approach, craniofacial SNP heritability was estimated as 65% for shape and 72% for size [12].

Using a pedigree of wild caught mice and a 2D approach, the heritability of mandible shape and size was found to be 0.61 and 0.49, respectively (Siahasarvie and Claude, personal communication). Regardless of the method or the experimental design, the heritability estimates for mandible size and shape in mice are high. It remains to be seen if the same pattern is true for the skull; pedigree-derived data need to be collected.

In a recent study of wild soay sheep, the SNP heritability of mandible length was estimated to be 53% [91]. Human studies estimate a narrow sense heritability of ~0.8 for facial morphology [18]. Although more data are needed, a pattern emerges from these studies: the form (i.e. shape and size) of craniofacial structures is a highly heritable trait.

### Candidate genes

The resolution achieved here was much higher than the resolution from traditional F_2_ crosses. Although the resolution was still not high enough to conclusively pinpoint individual genes, it was nonetheless possible to explore all genes within the QTL regions and often we identified a single candidate gene for which previous relevant phenotypic information existed. Moreover, 77% of the regions overlap with previous studies (see Tables 1 and 2); 7 of them with QTL regions derived from a backcross [10], 19 of them overlap with some of the ~ 4,000 enhancers active during craniofacial development in the mouse [11], and 1 region overlaps with a GWAS using a wild-derived population of mice [12]. Such overlaps cannot be explained by chance only (S3 Fig). Enhancers are DNA sequences that positively regulate the expression of nearby genes; therefore the high overlap with the enhancer dataset could indicate that some of the SNPs identified in this study tag a causal variant located in the regulatory region of the candidate genes.

The possibility of representing visually the shape traits associated with each SNP (see Figs 4 and S4–S8) allows the identification of specific craniofacial regions affected by the candidate genes. This information will become very valuable in future studies exploring the developmental role of such genes in craniofacial shape determination.

Many of the candidate genes are genes with reported craniofacial phenotypes. However, most of them were previously not quantitatively assessed and therefore knowledge of their specific effects on craniofacial shape variation requires a geometric morphometrics analysis of mutant mice. Such an analysis can be done in heterozygous knockout mice for the gene of interest. In this way the genetic alterations and their phenotypic effects are less drastic compared to the knockout of both alleles, and therefore closer to natural variability within populations [92]. Several other genes are new candidates for craniofacial shape determination; they are involved in diverse processes of bone formation but have not been directly implicated in craniofacial development.

We found two pairs of genes involved in the same signaling pathway; *Sh3pxd2b* and *Gab1* are part of the epidermal growth factor signaling pathway–EGF; *Sh3pxd2b* regulates EGF-mediated cell migration [78], and *Gab1* is involved in EGF-mediated cell growth [52]. *Mn1* and *Cldn18* are involved in the RANK-RANKL-OPG signaling pathway; *Mn1* regulates RANKL expression by stimulating RANKL’s promoter [66], and *Cldn18* regulates RANKL-induced differentiation of osteoclasts [35].

Among the candidate genes, *Mn1* is a particularly interesting one. It was originally discovered for being involved in a myeloid leukemia phenotype and it was therefore named *meningioma 1* [93]. This gene has the largest effect size in our screen (Tables 1 and 2) and is associated with many PCs in the skull and in the mandible (regions 4, 8, 12 in Table 1, regions 1 and 2 in Table 2, and S11 Fig), thus being also the most pleiotropic gene in our study. Knockout studies of *Mn1* revealed that the leukemia phenotype of the gene is only a by-product of a particular fusion with another gene, while the core function of *Mn1* lies in regulating the development of membranous bones of the cranial skeleton [65]. Intriguingly, *Mn1* is an orphan gene specific to bony vertebrates (Euteleostomi) (S12 Fig), a taxon characterized by the formation of bones and a suture-structured head skeleton. The origin of such orphan genes is connected to the emergence of evolutionary novelties [94], and the *Mn1* knockout phenotype in mouse suggests that it plays a crucial function in the emergence of a vertebrate novelty–the bony head. Hence, *Mn1* has the hallmarks of a key gene in the genetic architecture of craniofacial development and shape determination. The fact that it also emerges out of our genome-wide analysis lends credence to the notion that the approach is suitable to detect relevant genes even for highly polygenic phenotypes.

### Conclusions

There are long standing discussions about how to deal experimentally with polygenic traits and their implications for understanding the evolution of such traits [95,96]. Genome-wide association studies have certainly moved us forward in this respect. Even relatively simple quantitative phenotypes like human height have a highly polygenic nature [97,98]. Still, when a sufficiently powerful experimental design is used, key regulatory pathways influencing this phenotype can be identified [97,98]. The natural variants of these pathways have individually small effects, but knockouts of these genes can have large effects.

Here we have shown that we have a similar scenario regarding craniofacial shape, which is a complex phenotype with a highly polygenic architecture. It is encouraging to see that even under such seemingly adverse genetic conditions, we can still identify credible candidate genes previously studied in loss of function experiments. This implies that genes occupying central positions in developmental pathways may also be the ones that carry enough natural variation to allow mapping through GWAS. At the same time we identified regions without any previous information related to craniofacial development (skull regions 9 and 14; mandible regions 3, 5, 8 and 9); such discoveries contribute new information to the genetics underlying skull and mandible shape determination, and dedicated efforts should be made to understand the phenotypic effect of the genes and regulatory elements falling in such regions.

Human studies required ~25,000 individuals to explain 3–5% of height variation with genome-wide-significant SNPs [99], and ~250,000 to explain 16% [97]. Using ~5,400 individuals, only 5 loci were significantly associated with facial morphology in humans [18]. We have explained 4–11% of craniofacial variation using only ~700 outbred mice. Given the development of semi-automatic tools to speed up the phenotyping of shape traits (e.g. [100]), it seems feasible to increase both the number of animals involved, as well as to apply these tools to different mapping contexts. Hence, we are becoming more confident that an understanding of the biology behind craniofacial development will become possible.

## Materials and Methods

### Ethics statement

All procedures were approved by the University of Chicago Institutional Animal Care and Use Committee (IACUC) in accordance with National Institute of Health guidelines for the care and use of laboratory animals.Mapping population Male mice from the CFW mouse colony, maintained by Charles River Laboratories, were used for genome-wide association mapping. Upon their arrival at the University of Chicago, the mice were subjected to behavioral and physiological tests over the course of 2011 and 2012 (additional phenotype data from these tests are included in a separate manuscript that is being prepared for publication). At the end of these experiments, the mice were sacrificed and their heads were stored in ethanol. The average age at the time of sacrifice was 13 weeks (ranging from 12 to 14 weeks). Skulls and mandibles were measured in a subset of 720 mice between 2013 and 2014 at the Max Planck Institute for Evolutionary Biology in Plön, Germany.

### Shape phenotyping

Mouse heads were scanned using a computer tomograph (micro-CT—vivaCT 40; Scanco, Bruettisellen, Switzerland) at a resolution of 48 cross-sections per millimeter. Using the TINA landmarking tool [101], 44 three-dimensional landmarks were positioned in the skull, and 13 in each hemimandible (S1 Fig and S1 Table). The semi-automatic landmark annotation extension implemented in the TINA landmarking tool was used to reduce digitation error and accelerate the phenotyping process [100]. The raw 3D landmark coordinates obtained in TINA tool were exported to MorphoJ [102] for further morphometric analyses.

The symmetric component of the mandible and skull were obtained following [103]. In short, for mandible a full generalized Procrustes analysis (GPA) was performed with the landmark configurations of the right and left hemimandibles. The GPA eliminates the variation due to size, location, and orientation of the specimens, and generates a new dataset that only contains shape variation. For each individual, we recorded an average of the right and left resulting configurations, which represents the symmetric component of shape variation. For skull, a mirror image of the landmark configuration of each individual was generated, and a full GPA was performed with the original and mirror configurations. Again, the resulting configurations were averaged to obtain the symmetric component of shape variation. The new landmark coordinates generated by the GPA are called “Procrustes coordinates”.

To define shape features, we computed in MorphoJ principal components (PCs) from the n x 3k covariance matrix of Procrustes coordinates, where n is the number of samples and k is the number of landmarks; 3k represents the number of Procrustes coordinates (n = 590, k = 13 for mandible, and n = 710, k = 46 for skull). PC loadings computed in this analysis define the phenotypes used in the QTL mapping. Differences in age, spanning 2 weeks, did not correlate significantly with shape variation, so we did not use age as a covariate in subsequent analyses.

### Bone-mineral density (BMD)

In a separate project that will be presented in more detail in a later publication, areal BMD (aBMD) of the isolated femur was examined. Unexpectedly, we found that CFW mice appear to be predisposed toward abnormally high aBMD. This is a characteristic of the CFW mice that does not appear to be shared with commonly used inbred lab strains. A qualitative analysis of mice with high BMD showed substantial differences in mandible, and modest differences in the skull compared to mice with normal BMD. We therefore assessed covariation of BMD with shape measurements, separately for the skull and the mandible.

For the skull, we found a small correlation between BMD and shape (r^2^ = 1.4%, p(10,000 permutations) < 0.001). However, no individual PC corresponds to these shape differences due to BMD. Therefore, BMD was not used as covariate for skull trait mapping since it would have little to no effect on our ability to map QTLs for skull shape.

For the mandible, there was a stronger correlation between shape and BMD (r^2^ = 6%, p(10,000 permutations) <0.001). BMD accounts for 29% of the variation in the first PC, 8% of the variation in the third PC, but little to no variation in the remaining PCs (maximum r^2^ is 1.4% for PC6). Therefore, we computed mandible shape residuals by removing the linear effect of BMD; we used these residuals as input to the PCA, then PC loadings from this PCA analysis were used as phenotypes in the QTL mapping for mandible shape.

### Size phenotyping

The standard measure of size in geometric morphometrics is the centroid size (CS). This is the measure we used for mapping. Centroid size is defined as the square root of the sum of the squared distances of a set of landmarks from the center of gravity or centroid [104]. The CS for mandible was defined as the average of the CS of right and left hemimandible. The skull CS was calculated using all landmarks from right and left sides [103]. All these calculations were done in MorphoJ.

### Genotyping

The mice were genotyped using a genotyping-by-sequencing (GBS) approach [105]. In separate work, we have shown that GBS protocols can be used in combination with existing mouse genomics resources and software toolkits to obtain high-quality genotype data at a large number of genetic markers. In short, GBS libraries were prepared by digesting genomic DNA with the restriction enzyme *PstI* and annealing barcoded oligonucleotide adapters to the resulting overhangs. Samples were multiplexed 12 per lane, and sequenced on an Illumina HiSeq 2500 using single-end 100-bp reads. By focusing the sequencing effort on the *Pstl* restriction sites, we obtained high coverage at a subset of genomic loci. The 100-bp single-end reads were aligned to the Mouse Reference Assembly 38 from the NCBI database (mm10) using bwa [106]. We used a GBS-adapted version of the “best practices” pipeline of GATK [107–109] to discover variants and call genotypes. For the Variant Quality Score Recalibration (VQSR) step, we calibrated variant discovery against (1) whole-genome sequencing (WGS) data ascertained from a small set of CFW mice, (2) SNPs and indels from the Wellcome Trust Sanger Mouse Genome project [110], and SNPs available in dbSNP release 137.

GBS yields highly variable coverage across samples at the same cut site, hence variants with highly variable genotyping call rates. Therefore, to augment the set of SNPs with available genotypes, we used IMPUTE2 [111] to estimate missing genotypes and improve low confidence genotype calls. In total, we identified 92,374 autosomal SNPs.-

For QTL mapping, we took an additional step to filter out SNPs with low "imputation quality" assessed by inspecting the IMPUTE2 genotype probabilities (more precisely, any SNP in which less than 95% of the samples have a maximum probability genotype greater than 0.5), and SNPs with minor allele frequencies less than 2%. After completing this filtering step, we ended up with a final panel of 80,027 SNPs used to map QTLs on autosomal chromosomes.

### QTL mapping

720 mice were used for mapping loci associated with skull traits (shape and size). Due to the correlation between BMD and mandible shape, to assess support for mandible QTLs we used only the 592 mice for which BMD measurements were available.

We mapped QTLs for all PCs explaining at least 1% of total phenotypic variation in the sample; this includes 22 PCs capturing 84% of skull shape variation, and 21 PCs capturing 94% of mandible shape variation. Each PC was analyzed separately. To map size variation, the centroid size of mandible and skull was used. Note that the use of PCs restricts the findings to SNPs associated with the shape directions represented by such PCs; therefore genetic variants not aligned with the PC directions will not be detected with this approach.

We used the linear mixed model (LMM) implemented in GEMMA [112] to map the phenotypes, and at the same time to correct for the residual population structure that might still be present in the mapping population. The support for association with a given SNP is based on the p-value calculated from the likelihood-ratio test in GEMMA.

“Proximal contamination” refers to the loss in power to detect a QTL when the causal marker is included in the calculations of the kinship matrix [113,114]. In human genome-wide association studies with smaller sample sizes, this loss in power is expected to be minor [115]. However, in this study we expect that proximal contamination will have a larger impact on the genome-wide association analysis, particularly for genetic variants with larger effects, due to extended patterns of linkage disequilibrium in the CFW mouse population. To address this reduction of power due to proximal contamination, we took a ‘leave one chromosome out’ approach in which each chromosome is analyzed using a kinship matrix defined using all SNPs except SNPs on the chromosome being scanned [114,116].

A genome-wide significance threshold was calculated separately for each of the phenotypes used in the mapping (43 PCs and centroid size). A commonly used approach for assessing significance is to estimate the null distribution of p-values by randomly permuting the phenotype observations while keeping the genotypes the same. Such a procedure is technically not appropriate here because it fails to account for the lack of exchangeability among the samples, sometimes resulting in inflation of false positives [117,118]. However, since cryptic relatedness appears to have a small impact on association tests, a naive permutation test that assumes independence of the samples should provide an acceptable means to estimate the rate of false positive associations. This approach is supported by previous experiments we have performed in advanced intercross lines showing that improperly accounting for hidden relatedness in the permutations still produces a reasonable estimate for the significance threshold, despite the fact that advanced intercross lines have complex patterns of familial relationships [116]. Therefore, individual phenotypes were permuted 1,000 times, the distribution of minimum p-values was calculated, and the significance threshold was defined as 95% of this distribution. The average 95^th^ percentile for all phenotypes was 8.9 x 10^−7^ (-log(p) of 6.04, ranging from 5.97 to 6.16). This average threshold is depicted in Fig 1, but the exact threshold calculated separately for each phenotype was used to determine significance of the associations.

The LD pattern around the significant SNPs was used to define the QTL regions. A correlation value r^2^ ≥ 0.8 between the “peak” SNP (SNP with the smallest p-value) and the neighboring SNPs was used to select SNPs belonging to the QTL region. Genes falling within the QTL region were investigated using the MGI database [119] and literature search to suggest interesting gene candidates. Note that the choice of QTL region is inherently arbitrary, and it is possible that causal gene variant(s) underlying the QTL are not found within the QTL region as it is defined here.

### Effect size of individual loci

Numerous SNPs were statistically associated with various PCs; however, the effect of the SNP might go beyond the specific PC and affect other aspects of shape. Once we have identified genetic associations with individual PCs, we estimate the effect of the SNP on total shape. This is accomplished by fitting a standard multivariate regression model to the shape vectors (3k Procrustes coordinates) and SNP genotypes. This multivariate regression was implemented in MorphoJ. We report effect size as the proportion of shape variance explained by the SNP.

### Overlap with previous studies

Overlap with genetic loci reported in previous studies was assessed by defining 500-Kb and 1-Mb windows around the “peak” SNP—that is, the SNP with the lowest *p*-value—in each of the 26 QTLs identified in this study. This window size was chosen to correspond to the mean size of the QTL regions, which is 0.89Mb (see above for the way QTL regions were defined). Once the “true” overlap was determined, 26 genomic regions of 500 Kb and 1 Mb were randomly chosen from the genome and the overlap with previous studies was re-calculated. This was repeated 1,000 times to exclude the possibility that the global pattern of overlap was due to chance (S3 Fig).

### SNP heritability

SNP heritability of skull and mandible shape and size were estimated under the null linear model in GEMMA. We showed previously that for these traits, the null model and the Bayesian model implemented in GEMMA yield similar estimates [12]. These heritability estimates are defined as the proportion of phenotypic variation that can be explained by the SNPs used in the mapping; this estimate is often called “SNP heritability” [32].

We used a weighted sum over all PCs to summarize the “total heritability” of craniofacial shape. Each of the weights in this average is given by the proportion of total variation in the original phenotype explained by the PC (S2 and S3 Tables). By averaging over the individual heritability estimates across selected PCs, this yields a scalar value representing SNP heritability of skull and mandible shape. Shape is inherently a multivariate trait, and different shape directions might have different heritabilities [**120**,**121**]. Here we are not interested in which directions are more heritable than others; our goal is to capture how additive genetic variance contributes to overall phenotypic variation. From this perspective, the “total heritability” value not only informs about the role of genetics in trait variation, but also allows for comparison with other studies provided that the shape data are projected onto the same PCs [**122**,**123**].

### Partitioning of genetic variance by chromosome

The proportion of phenotypic variation explained by each chromosome was calculated using the restricted maximum-likelihood analysis implemented in GCTA (Yang et al. 2011). The first 10 principal components of the kinship matrix were included as covariates. An individual REML analysis was done for each chromosome (option–reml–grm–qcovar). Due to the small sample size of this study (~700 mice) it is not possible to fit all the chromosomes at the same time, which results in an inflation of the individual chromosomal estimates. We therefore used the relative (dividing by the variation explain by all chromosomes together) and not the absolute contribution of each chromosome to the total phenotypic variation.

Because PCs were used as phenotypes, additional calculations were needed to estimate the chromosomal contribution to the global phenotypes–skull and mandible shape. The additive variance per chromosome per PC was multiplied by the proportion of phenotypic variation represented by that PC. Finally, the values for each chromosome were summed across all PCs.

### Software and data resources

The full code and data reproducing the steps of our analyses are available for download at http://dx.doi.org/10.5061/dryad.k543p.

## Supporting Information

S1 Table Description of skull and mandible landmark positions used in the geometric morphometrics analysis. (PDF)

S2 Table Principal components used to map QTLs for mandible shape. %var, portion of the total skull variation explained by each PC. %cum, cumulative variance explained by the PCs. PVE, proportion of variance in the PC explained by the SNPs used in the QTL mapping. se(PVE), standard error of PVE estimate. PCA was run in MorphoJ. PVE estimates were obtained from the LMM implemented in GEMMA.
(PDF)

S3 Table Principal components used to map QTLs for skull shape. %var, portion of the total skull variation explained by each PC. %cum, cumulative variance explained by the PCs. PVE, proportion of variance in the PC explained by the SNPs used in the QTL mapping. se(PVE), standard error of PVE estimate. PCA was run in MorphoJ. PVE estimates were obtained from the LMM implemented in GEMMA.
(PDF)

S4 Table Regions associated with skull shape variation. The intervals were defined by LD blocks around the peak SNP using a threshold of r2 > = 0.8 with the peak SNP. The genes within the QTL regions are listed in this table. When it was not possible to define regions due to a sparse LD signal, the gene overlapping the peak SNP is shown (*), or genes close to the peak SNP that could be considered candidate genes. Where no gene is shown, no genes meeting the above criterion were identified at the locus. The gene (genebody in Attanasio *et al* 2014) associated with the closer enhancer to the peak SNP is shown (**).
(PDF)

S5 Table Regions associated with mandible shape variation. The intervals were defined by LD blocks around the peak SNP using a threshold of r2 > = 0.8 with the peak SNP. The genes within the QTL regions are listed in this table. When it was not possible to define regions due to a sparse LD signal, the gene overlapping the peak SNP is shown (*), or genes close to the peak SNP that could be considered candidate genes. Where no gene is shown, no genes meeting the above criterion were identified at the locus. The gene (genebody in Attanasio *et al* 2014) associated with the closer enhancer to the peak SNP is shown (**).
(PDF)

S1 Fig Landmark points used to measure skull and mandible shape. See description of the points in S1 Table.
(PDF)

S2 Fig Marker coverage of the genome. The number of SNPs per autosomal chromosome are shown.
(PDF)

S3 Fig Overlap with previous studies. 26 genomic regions were chosen randomly and their overlap with previous studies was calculated, this was repeated 1000 times. (a) overlap between 1-Mb regions and the results from Attanasio *et al* 2014. (b) overlap between 500-Kb regions and Attanasio *et al* 2014. (c) overlap of 1-Mb regions and Maga *et al* 2015.
(PDF)

S4 Fig Two-dimensional representation of the 3D changes in skull shape associated to the significant SNPs. SNPs rs258942042, rs245694506, rs228570244, and rs30491142 are shown. In grey (dotted lines) is the sample mean of mandible shape. In orange (continuous line) is the mean shape represented by the regression vector of skull shape on SNP genotype (scale, 10x). Lateral, dorsal, frontal, and ventral views, as well as the relevant landmarks (solid dots) for each view are shown.
(PDF)

S5 Fig Two-dimensional representation of the 3D changes in skull shape associated to the significant SNPs. SNPs rs27194486, rs13466556, rs254983846, and rs37378594 are shown. In grey (dotted lines) is the population’s mean mandible shape. In orange (continuous line) is the shape represented by the regression vector of skull shape on SNP genotype (scale, 10x). Lateral, dorsal, frontal, and ventral views, as well as the relevant landmarks (solid dots) for each view are shown.
(PDF)

S6 Fig Two-dimensional representation of the 3D changes in skull shape associated to the significant SNPs. SNPs cfw-13-31734894, rs30352013, rs227631022, and rs26862534 are shown. In grey (dotted lines) is the population’s mean mandible shape. In orange (continuous line) is the shape represented by the regression vector of skull shape on SNP genotype (scale, 10x). Lateral, dorsal, frontal, and ventral views, as well as the relevant landmarks (solid dots) for each view are shown.
(PDF)

S7 Fig Two-dimensional representation of the 3D changes in skull shape associated to the significant SNPs. SNPs rs26992385, rs46747509, rs50079241, and rs31584944 are shown. In grey (dotted lines) is the population’s mean mandible shape. In orange (continuous line) is the shape represented by the regression vector of skull shape on SNP genotype (scale, 10x). Lateral, dorsal, frontal, and ventral views, as well as the relevant landmarks (solid dots) for each view are shown.
(PDF)

S8 Fig Two-dimensional representation of the 3D changes in mandible shape associated to the significant SNPs. SNPs rs33217671, rs28219152, rs29977169, rs36343125, rs237064333, rs233696367, and rs221759350 are shown. In grey (dotted lines) is the population’s mean mandible shape. In orange (continuous line) is the shape represented by the regression vector of skull shape on SNP genotype (scale, 10x). Lateral, dorsal, frontal, and ventral views, as well as the relevant landmarks (solid dots) for each view are shown.
(PDF)

S9 Fig Genome-wide scans for mandible PCs. Only the PCs with significant associations are shown.
(PDF)

S10 Fig Genome-wide scans for skull PCs. Only the PCs with significant associations are shown.
(PDF)

S11 Fig Regional plot of the association signal around the *Mn1* gene. Genomic regions associated with (a) mandible shape, and (b) skull shape are shown. The SNP with lowest p-value is labeled and shown in purple. Dots represent neighboring SNPs; color indicates LD (r2) with the labeled SNP.
(PDF)

S12 Fig Origin of *Mn1* at the base of Eutelostomi. (a) Using PSI-BLAST at NCBI (http://blast.ncbi.nlm.nih.gov/Blast.cgi?CMD=Web&PAGE_TYPE=BlastHome) with 3 iterations and a threshold of 0.005 (low complexity filter activated), no matches were found beyond bony fish. (b) Using genomic context analysis with Genomicus (http://www.genomicus.biologie.ens.fr/genomicus-81.01/cgi-bin/search.pl) hits were detected only in Euteleostomi.
(PDF)
