# Supplementary material for: Mapping of Craniofacial Traits in Outbred Mice Identifies Major Developmental Genes Involved in Shape Determination
Source: PLoS Genet. 2015 Nov 2;11(11):e1005607. doi: 10.1371/journal.pgen.1005607 (PMC4629907; doi:10.1371/journal.pgen.1005607)
Supplement: S1 Table — (PDF) [file pgen.1005607.s001.pdf]

Table S1. Description of skull and mandible landmark positions used in the geometric morphometrics analysis.

Landmark	Description
**Skull**	
**1**	Nasal bones most anterior suture
**2**	Nasal bones most posterior suture
**3**	Frontal bones most posterior suture
**4**	Parietal bones most posterior suture
**5**	Interparietal bone most posterior point on the median line
**6**	Right side, most anterior point of the suture between frontal and parietal bones
**7**	Left side, most anterior point of the suture between frontal and parietal bones
**8**	Right side, intersection between parietal, occipital and squamosal bones
**9**	Left side, intersection between parietal, occipital and squamosal bones
**10**	Right, most posterior junction of squamosal bone and the zygomatic process of the squamosal bone
**11**	Left, most posterior junction of squamosal bone and the zygomatic process of the squamosal bone
**12**	Right side, most anterior suture of the zygomatic process of the squamosal bone and jugal bone
**13**	Left side, most anterior suture of the zygomatic process of the squamosal bone and jugal bone
**14**	Right side, most anterior suture of jugal bone and the zygomatic process of the maxillary bone
**15**	Left side, most anterior suture of jugal bone and the zygomatic process of the maxillary bone
**16**	Right side, intersection of the frontal, lacrimal and the zygomatic process of the maxillary bone
**17**	Left side, intersection of the frontal, lacrimal and the zygomatic process of the maxillary bone
**18**	Right infraorbital foramen most superior point
**19**	Left infraorbital foramen most superior point
**20**	Right infraorbital foramen most inferior point
**21**	Left infraorbital foramen most inferior point
**22**	Right premaxilla-right nasal bone most anterior point of suture
**23**	Left premaxilla-left nasal bone most anterior point of suture
**24**	Most superior point of the right incisor alveolus
**25**	Most superior edge of the left incisor alveolus
**26**	Most inferior point of the right incisor alveolus
**27**	Most inferior point of the left incisor alveolus
**28**	Right premaxilla-maxilla most ventral juntion
**29**	Left premaxilla-maxilla most ventral juntion
**30**	Most anterior point of the right first molar alveolus
**31**	Most anterior point of the left first molar alveolus
**32**	Most posterior point of the right third molar alveolus
**33**	Most posterior point of the left third molar alveolus
**34**	Most anterior point of the right anterior palatine foramen
**35**	Most anterior point of the left anterior palatine foramen
**36**	Most posterior point of the right anterior palatine foramen
**37**	Most posterior point of the left anterior palatine foramen
**38**	Right pterygoid process, most posterior point
**39**	Left pterygoid process, most posterior point
**40**	Median-line point of the suture between occipital and basisphenoid bones
**41**	Median-line point of the suture between basisphenoid and presphenoid bones
**42**	Most posterior point of the suture between palatine bones
**43**	Foramen magnum most anterior point, Basion
**44**	Foramen magnum most posterior point, Bregma
**Mandible**	
**1**	Most superior point of the incisor alveolus
**2**	Most inferior point of the incisor alveolus
**3**	Most anterior point of the first molar alveolus
**4**	Most posterior point of the third molar alveolus
**5**	Most posterior tip of the coronoid process

**6**	Most anterior concave point of coronoid process
**7**	Most anterior point of the articular surface of the condyle
**8**	Most posterior tip of the condyle
**9**	Most anterior concave point between condyle and angular process
**10**	Most posterior tip of angular process
**11**	Most inferior point of angular process
**12**	Ascending ramus dorsal-most ventral point
**13**	Alveolar region most inferior point
