# Supplementary material for: Mapping of Craniofacial Traits in Outbred Mice Identifies Major Developmental Genes Involved in Shape Determination
Source: PLoS Genet. 2015 Nov 2;11(11):e1005607. doi: 10.1371/journal.pgen.1005607 (PMC4629907; doi:10.1371/journal.pgen.1005607)
Supplement: S3 Table — %var, portion of the total skull variation explained by each PC. %cum, cumulative variance explained by the PCs. PVE, proportion of variance in the PC explained by the SNPs used in the QTL mapping. se(PVE), standard error of PVE estimate. PCA was run in MorphoJ. PVE estimates were obtained from the LMM implemented in GEMMA. (PDF) [file pgen.1005607.s003.pdf]

Table S3. Principal components used to map QTLs for skull shape. %var, portion of the total skull variation explained by each PC. %cum, cumulative variance explained by the PCs. PVE, proportion of variance in the PC explained by the SNPs used in the QTL mapping. se(PVE), standard error of PVE estimate. PCA was run in MorphoJ. PVE estimates were obtained from the LMM implemented in GEMMA.

PC	%var	%cum	PVE	se(PVE)
**1**	15.7	15.7	66.2	9.8
**2**	9.5	25.2	52.8	10.7
**3**	8.4	33.6	66.9	8.1
**4**	5.1	38.7	36.3	9.3
**5**	4.7	43.4	51.8	9.4
**6**	4.5	47.9	58.9	8.8
**7**	4.1	52.0	46.9	9.6
**8**	4.0	56.0	42.0	10.6
**9**	3.5	59.5	40.7	9.3
**10**	3.0	62.5	26.9	10.2
**11**	2.9	65.3	36.3	9.4
**12**	2.5	67.8	45.0	10.6
**13**	2.3	70.1	61.5	10.6
**14**	2.2	72.3	29.7	9.9
**15**	2.0	74.3	14.0	8.6
**16**	1.8	76.0	43.0	9.9
**17**	1.6	77.7	47.3	9.1
**18**	1.4	79.1	42.1	9.9
**19**	1.3	80.3	55.2	9.1
**20**	1.2	81.6	58.2	9.0
**21**	1.2	82.8	51.2	9.3
**22**	1.1	83.8	38.5	9.4
**SNP heritability of skull shape**			42.44	
**SNP heritability of skull size**			35.4	10.4
