# Supplementary material for: Mapping of Craniofacial Traits in Outbred Mice Identifies Major Developmental Genes Involved in Shape Determination
Source: PLoS Genet. 2015 Nov 2;11(11):e1005607. doi: 10.1371/journal.pgen.1005607 (PMC4629907; doi:10.1371/journal.pgen.1005607)
Supplement: S4 Table — The intervals were defined by LD blocks around the peak SNP using a threshold of r2 > = 0.8 with the peak SNP. The genes within the QTL regions are listed in this table. When it was not possible to define regions due to a sparse LD signal, the gene overlapping the peak SNP is shown (*), or genes close to the peak SNP that could be considered candidate genes. Where no gene is shown, no genes meeting the above criterion were identified at the locus. The gene (genebody in Attanasio et al 2014) associated with the closer enhancer to the peak SNP is shown (**). (PDF) [file pgen.1005607.s004.pdf]

Table S4. Regions associated with skull shape variation. The intervals were defined by LD blocks around the peak SNP using a threshold of r2 >=0.8 with the peak SNP. The genes within the QTL regions are listed in this table. When it was not possible to define regions due to a sparse LD signal, the gene overlapping the peak SNP is shown (*), or genes close to the peak SNP that could be considered candidate genes. Where no gene is shown, no genes meeting the above criterion were identified at the locus. The gene (genebody in Attanasio *et al* 2014) associated with the closer enhancer to the peak SNP is shown (**).

Region	Skull	Interval	Genes
**1**	PC1	chr11	Sh3pxd2b*
**2**	PC1	chr13	Rab3c*, Plk2, Pde4d**
**e**	PC2	chr8:80366708-82427186	Gypa, Frem3, Smarca5, Il15, Gab1, Usp38, Inpp4b
**4**	PC3	chr5:111215141-111426493	Pitpnb, Mn1, Ttc28**
**5**	PC4	chr9:99662257-99713529	Cldn18, Dzip1l (4930519F24Rik**)
**6**	PC5	chr2:33284278-34883623	Hspa5, Zbtb43, Lmx1b**, Rabepk, Fbxw2, Cutal, Ralgps1, Zbtb34, Mvb12b, Pbx3, Psmd5, Gapvd1, Mapkap1
**7**	PC5	chr9	Copb2*, Foxl2**
**8**	PC6	chr5:111626960-112398133	Srrd, Asphd2, Crybb1, Tpst2, Cryba4, Hps4, Tfip11 (C130026L21Rik**)
**r**	PC6	chr19:4001698-4180585	Nudt8, Doc2g, Ndufv1, Gstp2, Gstp1, BC021614, Cabp2, Cdk2ap2, Aip, Cabp4, Ptprcap, Coro1b, Carns1, Rps6kb2, Pitpnm1, Gpr152, Tmem134 (Kdm2a**)
**e**	PC7	chr13	Foxf2**, Foxc1
**11**	PC8	chr3:98278704-99786152	Hsd3b5, Hsd3b1, Phgdh, Zfp697, Hsd3b4, Gm4450, Hsd3b6, Hsd3b2, Hsd3b3, Hao2**, Hmgcs2, Wars2, Tbx15
**12**	PC8	chr5	Ttc28* **, Mn1
**13**	PC12	chr11	Sh3pxd2b*
**14**	PC19	chr11	(Phb**)
**15**	PC20	chr2:82522884-84982758	Btbd18, 2700094K13Rik, Med19, Clp1, Timm10, Prg2, Zc3h15, Fam171b, Zswim2, Tfpi, Ctnnd1, Serping1, Ypel4, Zdhhc5, Slc43a1, Smtnl1, Rtn4rl2, Slc43a3, Itgav, Fsip2, Calcrl, Ube2l6, Tmx2
**16**	PC20	chr11:94826955-95144609	Hils1, Sgca, Dlx3, Col1a1, Samd14, Ppp1r9b, Dlx4, Itga3, Pdk2 (Gm11544**)
**17**	PC22	chr15:11384042-12107575	Tars, Npr3, Sub1 (Adamts12**)
