# Supplementary material for: Mapping of Craniofacial Traits in Outbred Mice Identifies Major Developmental Genes Involved in Shape Determination
Source: PLoS Genet. 2015 Nov 2;11(11):e1005607. doi: 10.1371/journal.pgen.1005607 (PMC4629907; doi:10.1371/journal.pgen.1005607)
Supplement: S5 Table — The intervals were defined by LD blocks around the peak SNP using a threshold of r2 > = 0.8 with the peak SNP. The genes within the QTL regions are listed in this table. When it was not possible to define regions due to a sparse LD signal, the gene overlapping the peak SNP is shown (*), or genes close to the peak SNP that could be considered candidate genes. Where no gene is shown, no genes meeting the above criterion were identified at the locus. The gene (genebody in Attanasio et al 2014) associated with the closer enhancer to the peak SNP is shown (**). (PDF) [file pgen.1005607.s005.pdf]

Table S5. Regions associated with mandible shape variation. The intervals were defined by LD blocks around the peak SNP using a threshold of r2 >=0.8 with the peak SNP. The genes within the QTL regions are listed in this table. When it was not possible to define regions due to a sparse LD signal, the gene overlapping the peak SNP is shown (*), or genes close to the peak SNP that could be considered candidate genes. Where no gene is shown, no genes meeting the above criterion were identified at the locus. The gene (genebody in Attanasio *et al* 2014) associated with the closer enhancer to the peak SNP is shown (**).

Region	Mandible	Interval	Genes
**1**	PC4	chr5	Ttc28*, Mn1
**2**	PC7	chr5:111215141-111426493	Pitpnb, Mn1**, Ttc28
**3**	PC7	chr11	Slit3* **
**4**	PC8	chr9	Cldn18 (4930519F24Rik**)
**5**	PC12	chr6	-
**6**	PC15	chr14:98366257-99303998	Bora, Mzt1, Klf5, Pibf1, Dis3
**7**	PC19	chr11:96201066-96737262	Hoxb1-8, Skap1 (Igf2bp1**)
**8**	PC20	chr4	-
**9**	Centroid size	chr1	Dhx9* (Rgs8**)
