# Supplementary material for: Mapping of Craniofacial Traits in Outbred Mice Identifies Major Developmental Genes Involved in Shape Determination
Source: PLoS Genet. 2015 Nov 2;11(11):e1005607. doi: 10.1371/journal.pgen.1005607 (PMC4629907; doi:10.1371/journal.pgen.1005607)
Supplement: S1 Fig — See description of the points in S1 Table. (PDF) [file pgen.1005607.s006.pdf]

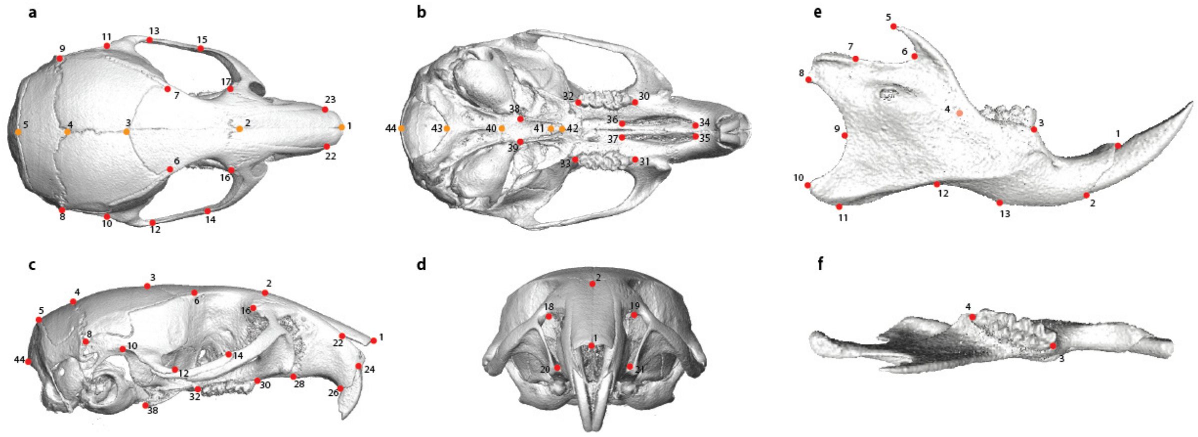

Figure S1. Landmark points used to measure skull and mandible shape. See description of the points in Table S1.
