# Supplementary figures and images for: Mapping of Craniofacial Traits in Outbred Mice Identifies Major Developmental Genes Involved in Shape Determination
Source: PLoS Genet. 2015 Nov 2;11(11):e1005607. doi: 10.1371/journal.pgen.1005607 (PMC4629907; doi:10.1371/journal.pgen.1005607)

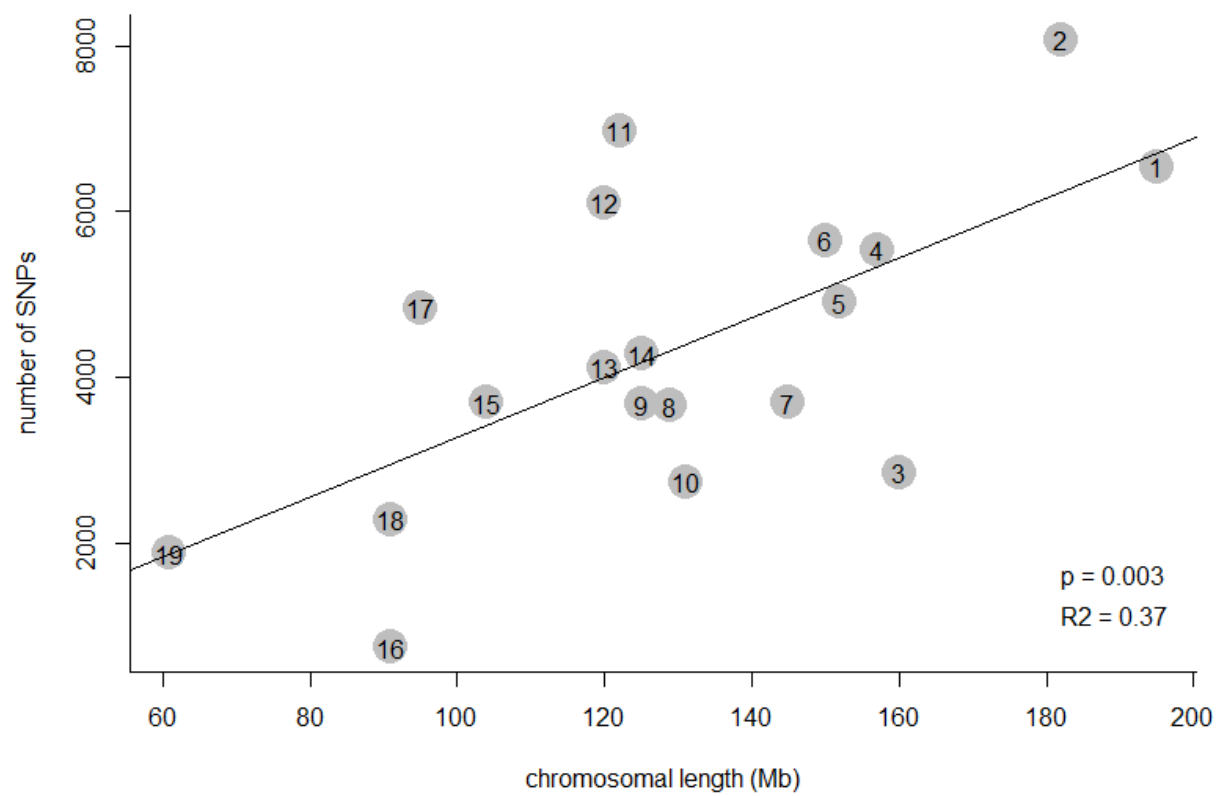

**Figure S2. Marker coverage of the genome.** The number of SNPs per autosomal chromosome are shown.

Supplement: S2 Fig — The number of SNPs per autosomal chromosome are shown. (PDF) [file pgen.1005607.s007.pdf]

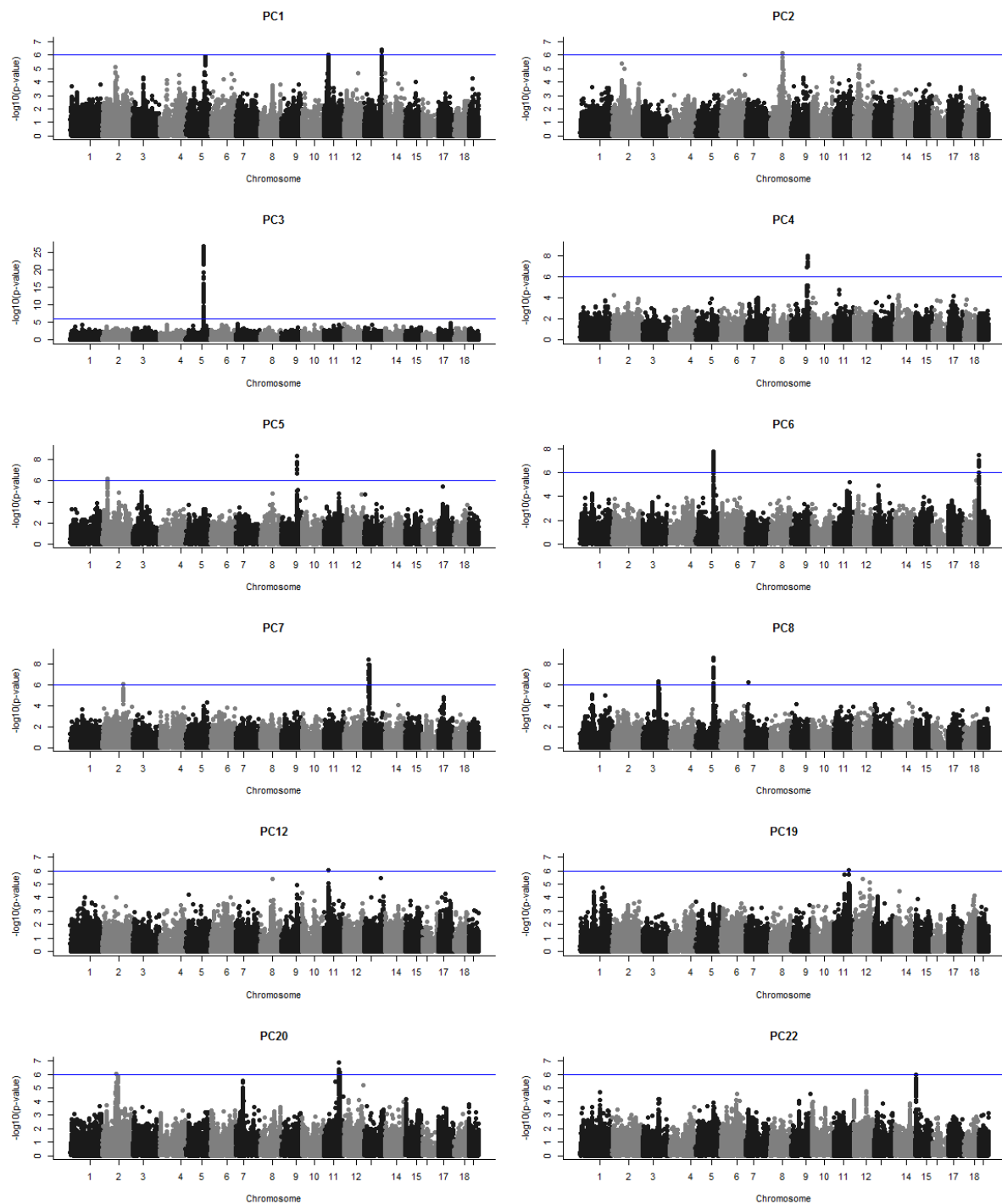

**Figure S10. Genome-wide scans for skull PCs.** Only the PCs with significant associations are shown.

Supplement: S10 Fig — Only the PCs with significant associations are shown. (PDF) [file pgen.1005607.s015.pdf]
