# Supplementary material for: Mapping of Craniofacial Traits in Outbred Mice Identifies Major Developmental Genes Involved in Shape Determination
Source: PLoS Genet. 2015 Nov 2;11(11):e1005607. doi: 10.1371/journal.pgen.1005607 (PMC4629907; doi:10.1371/journal.pgen.1005607)
Supplement: S3 Fig — 26 genomic regions were chosen randomly and their overlap with previous studies was calculated, this was repeated 1000 times. (a) overlap between 1-Mb regions and the results from Attanasio et al 2014. (b) overlap between 500-Kb regions and Attanasio et al 2014. (c) overlap of 1-Mb regions and Maga et al 2015. (PDF) [file pgen.1005607.s008.pdf]

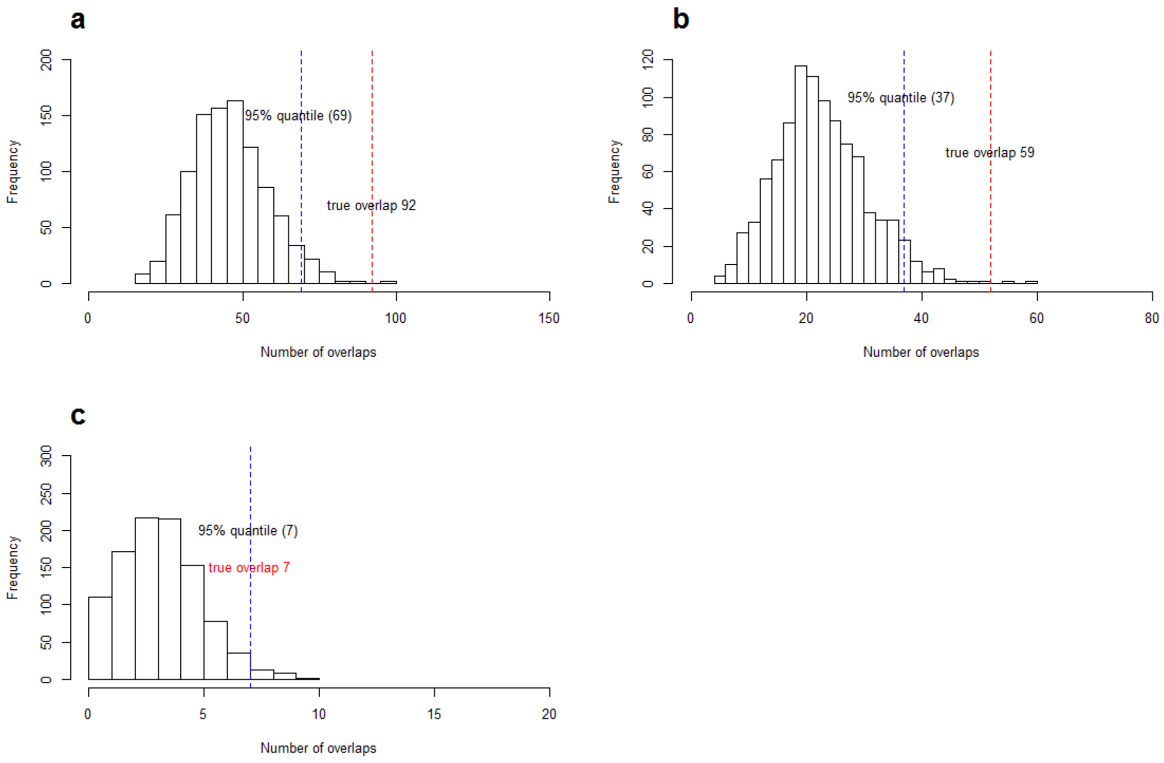

Figure S3. Overlap with previous studies. 26 genomic regions were chosen randomly and their overlap with previous studies was calculated, this was repeated 1000 times. (a) overlap between 1Mb regions and the results from Attanasio *et al* 2014. (b) overlap between 500 Kb regions and Attanasio *et al* 2014. (c) overlap of 1Mb regions and Maga *et al* 2015.
