# Supplementary material for: Mapping of Craniofacial Traits in Outbred Mice Identifies Major Developmental Genes Involved in Shape Determination
Source: PLoS Genet. 2015 Nov 2;11(11):e1005607. doi: 10.1371/journal.pgen.1005607 (PMC4629907; doi:10.1371/journal.pgen.1005607)
Supplement: S5 Fig — SNPs rs27194486, rs13466556, rs254983846, and rs37378594 are shown. In grey (dotted lines) is the population’s mean mandible shape. In orange (continuous line) is the shape represented by the regression vector of skull shape on SNP genotype (scale, 10x). Lateral, dorsal, frontal, and ventral views, as well as the relevant landmarks (solid dots) for each view are shown. (PDF) [file pgen.1005607.s010.pdf]

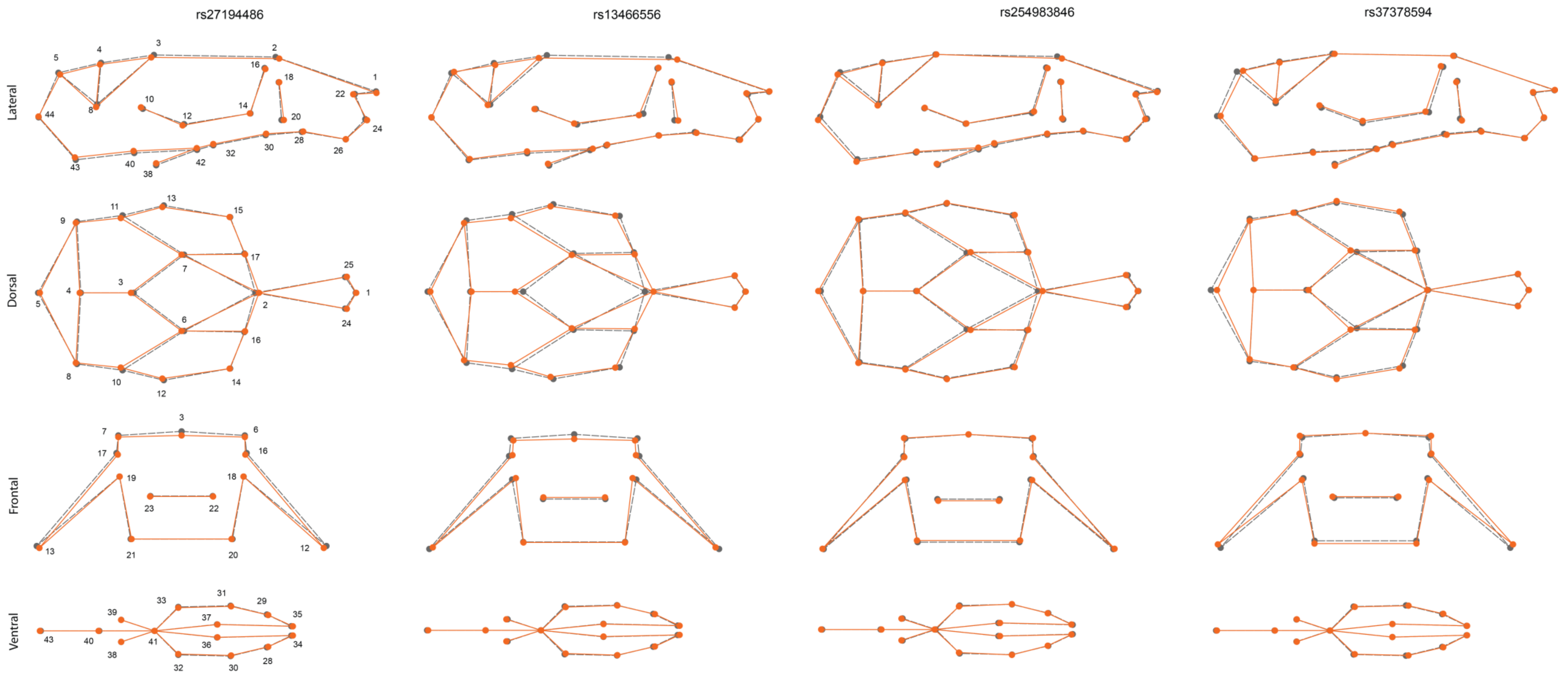

Figure S5. Two-dimensional representation of the 3D changes in skull shape associated to the significant SNPs. SNPs rs27194486, rs13466556, rs254983846, and rs37378594 are shown. In grey (dotted lines) is the population's mean mandible shape. In orange (continuous line) is the shape represented by the regression vector of skull shape on SNP genotype (scale, 10x). Lateral, dorsal, frontal, and ventral views, as well as the relevant landmarks (solid dots) for each view are shown.
