# Supplementary material for: Mapping of Craniofacial Traits in Outbred Mice Identifies Major Developmental Genes Involved in Shape Determination
Source: PLoS Genet. 2015 Nov 2;11(11):e1005607. doi: 10.1371/journal.pgen.1005607 (PMC4629907; doi:10.1371/journal.pgen.1005607)
Supplement: S9 Fig — Only the PCs with significant associations are shown. (PDF) [file pgen.1005607.s014.pdf]

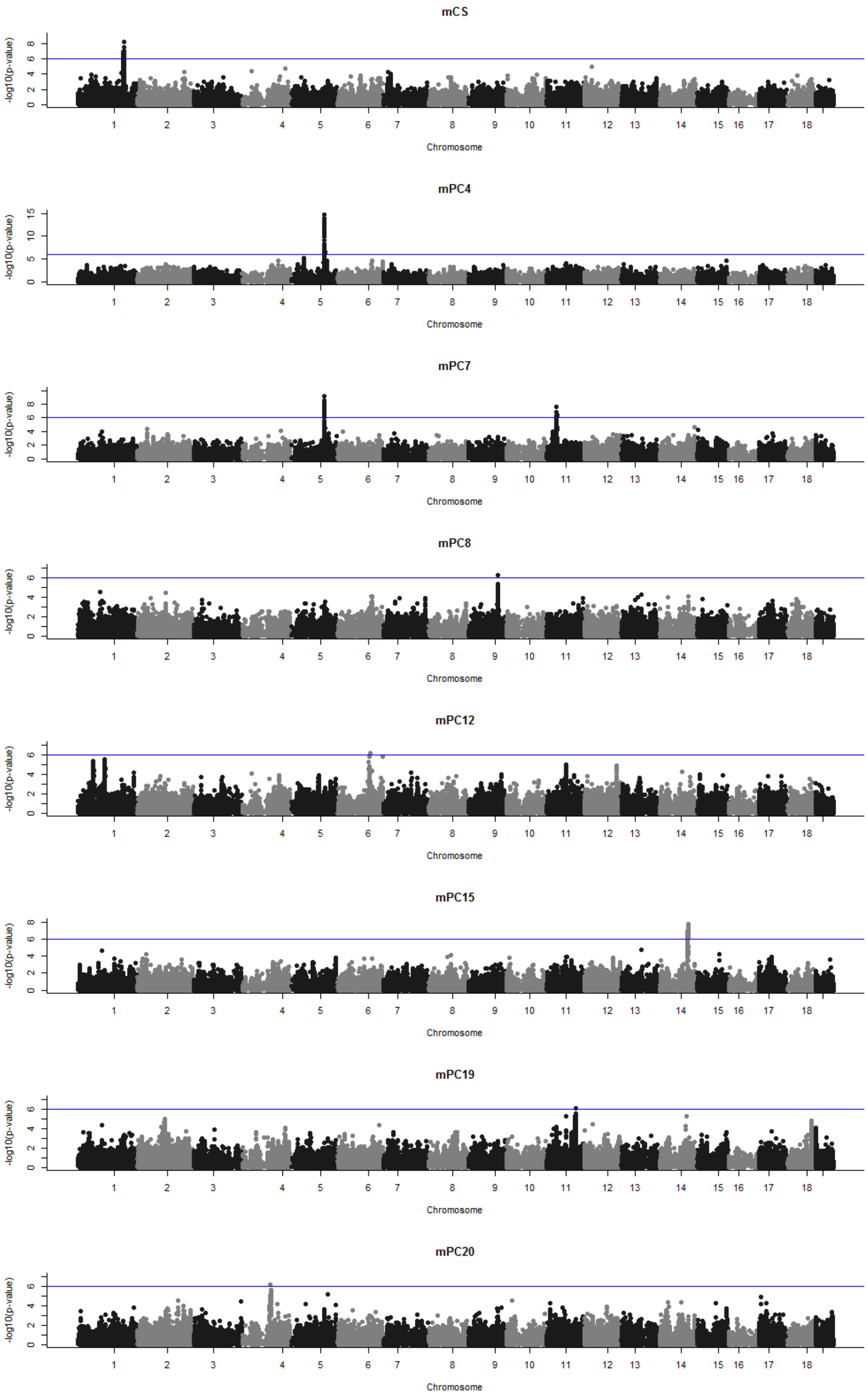

Figure S10. Genome-wide scans for mandible PCs. Only the PCs with significant associations are shown.
