# Supplementary material for: Mapping of Craniofacial Traits in Outbred Mice Identifies Major Developmental Genes Involved in Shape Determination
Source: PLoS Genet. 2015 Nov 2;11(11):e1005607. doi: 10.1371/journal.pgen.1005607 (PMC4629907; doi:10.1371/journal.pgen.1005607)
Supplement: S11 Fig — Genomic regions associated with (a) mandible shape, and (b) skull shape are shown. The SNP with lowest p-value is labeled and shown in purple. Dots represent neighboring SNPs; color indicates LD (r2) with the labeled SNP. (PDF) [file pgen.1005607.s016.pdf]

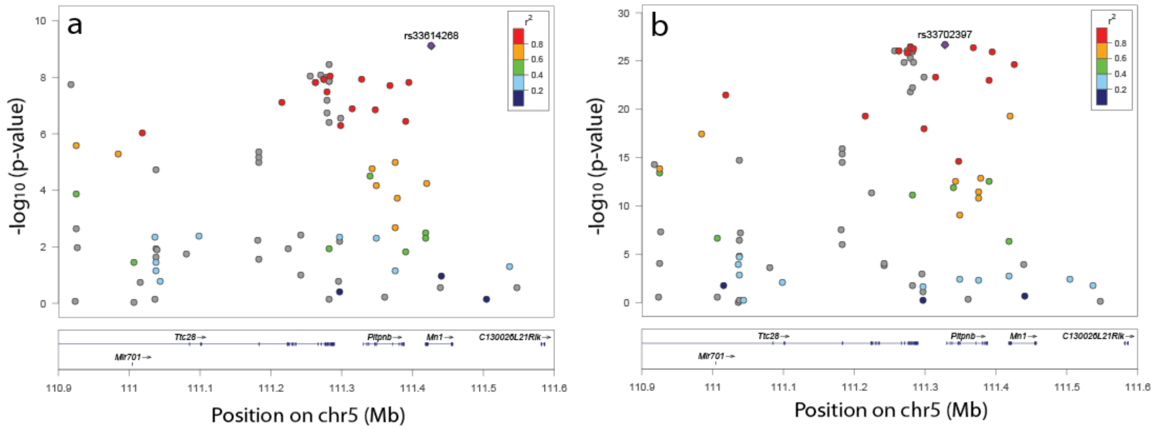

Figure S11. Regional plot of the association signal around the *Mn1* gene. Genomic regions associated with (a) mandible shape, and (b) skull shape are shown. The SNP with lowest p-value is labeled and shown in purple. Dots represent neighboring SNPs; color indicates LD (r^2) with the labeled SNP.
