# Supplementary material for: Mapping of Craniofacial Traits in Outbred Mice Identifies Major Developmental Genes Involved in Shape Determination
Source: PLoS Genet. 2015 Nov 2;11(11):e1005607. doi: 10.1371/journal.pgen.1005607 (PMC4629907; doi:10.1371/journal.pgen.1005607)
Supplement: S12 Fig — (a) Using PSI-BLAST at NCBI (http://blast.ncbi.nlm.nih.gov/Blast.cgi?CMD=Web&PAGE_TYPE=BlastHome) with 3 iterations and a threshold of 0.005 (low complexity filter activated), no matches were found beyond bony fish. (b) Using genomic context analysis with Genomicus (http://www.genomicus.biologie.ens.fr/genomicus-81.01/cgi-bin/search.pl) hits were detected only in Euteleostomi. (PDF) [file pgen.1005607.s017.pdf]

**a**

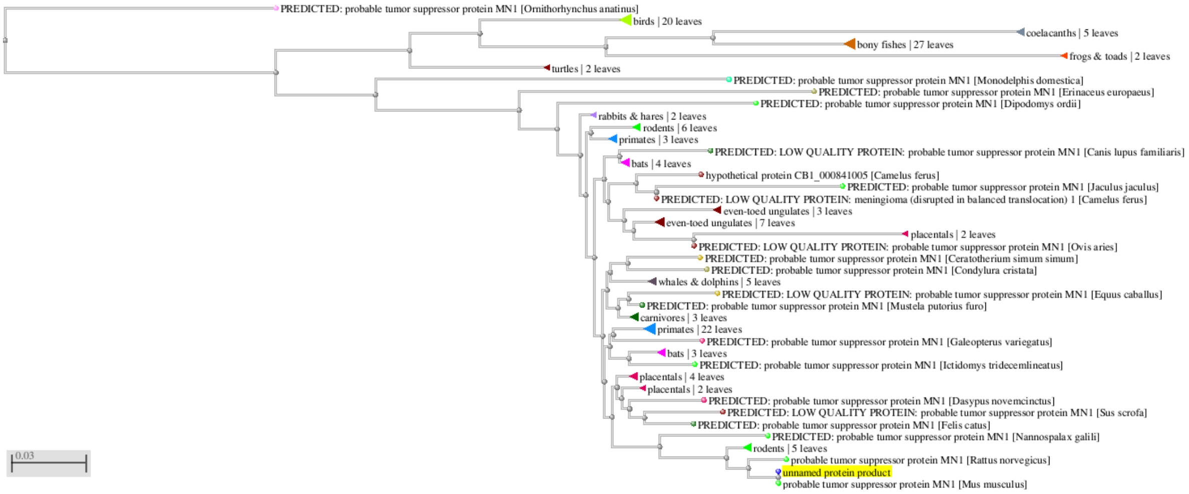


**b**

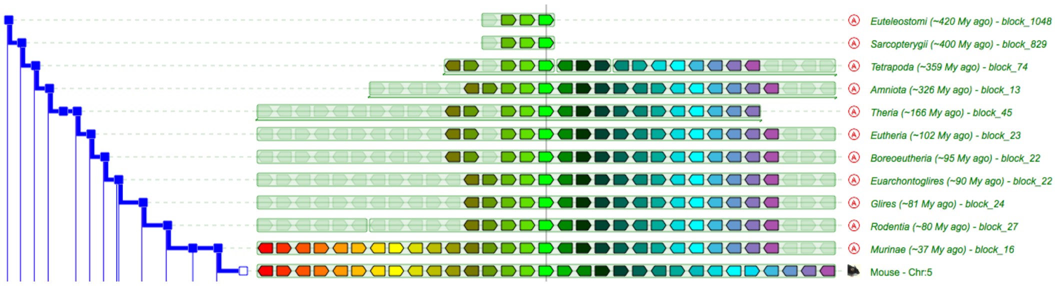


**Figure S12. Origin of Mn1 at the base of Eutelostomi.** (a) Using PSI-BLAST at NCBI (http://blast.ncbi.nlm.nih.gov/Blast.cgi?CMD=Web&PAGE_TYPE=BlastHome) with 3 iterations and a threshold of 0.005 (low complexity filter activated), no matches were found beyond bony fish. (b) Using genomic context analysis with Genomicus (http://www.genomicus.biologie.ens.fr/genomicus-81.01/cgi-bin/search.pl) hits were detected only in Euteleostomi
